# Active eukaryotes in drinking water distribution systems of ground and surface waterworks

**DOI:** 10.1186/s40168-019-0715-5

**Published:** 2019-07-03

**Authors:** Jenni Inkinen, Balamuralikrishna Jayaprakash, Sallamaari Siponen, Anna-Maria Hokajärvi, Anna Pursiainen, Jenni Ikonen, Ivan Ryzhikov, Martin Täubel, Ari Kauppinen, Jussi Paananen, Ilkka T. Miettinen, Eila Torvinen, Mikko Kolehmainen, Tarja Pitkänen

**Affiliations:** 1 Department of Health Security, National Institute for Health and Welfare, P.O. Box 95, FI-70701 Kuopio, Finland; 2 Institute of Biomedicine, University of Eastern Finland, P.O. Box 1627, FI-70211 Kuopio, Finland; 3 Department of Environmental and Biological Sciences, University of Eastern Finland, P.O. Box, 1627, FI-70211 Kuopio, Finland

**Keywords:** 18S rRNA, Eukaryotic communities, Drinking water distribution system, Active biota members, Disinfection, Hot water systems, Biofilms

## Abstract

**Background:**

Eukaryotes are ubiquitous in natural environments such as soil and freshwater. Little is known of their presence in drinking water distribution systems (DWDSs) or of the environmental conditions that affect their activity and survival.

**Methods:**

Eukaryotes were characterized by Illumina high-throughput sequencing targeting 18S rRNA gene (DNA) that estimates the total community and the 18S rRNA gene transcript (RNA) that is more representative of the active part of the community. DWDS cold water (*N* = 124), hot water (*N* = 40), and biofilm (*N* = 16) samples were collected from four cities in Finland. The sampled DWDSs were from two waterworks A–B with non-disinfected, recharged groundwater as source water and from three waterworks utilizing chlorinated water (two DWDSs of surface waterworks C–D and one of ground waterworks E). In each DWDS, samples were collected from three locations during four seasons of 1 year.

**Results:**

A beta-diversity analysis revealed that the main driver shaping the eukaryotic communities was the DWDS (A–E) (*R* = 0.73, *P* < 0.001, ANOSIM). The kingdoms *Chloroplastida* (green plants and algae), *Metazoa* (animals: rotifers, nematodes), *Fungi* (e.g., *Cryptomycota*), *Alveolata* (ciliates, dinoflagellates), and *Stramenopiles* (algae *Ochrophyta*) were well represented and active—judging based on the rRNA gene transcripts—depending on the surrounding conditions. The unchlorinated cold water of systems (A–B) contained a higher estimated total number of taxa (Chao1, average 380–480) than chlorinated cold water in systems C–E (Chao1 ≤ 210). Within each DWDS, unique eukaryotic communities were identified at different locations as was the case also for cold water, hot water, and biofilms. A season did not have a consistent impact on the eukaryotic community among DWDSs.

**Conclusions:**

This study comprehensively characterized the eukaryotic community members within the DWDS of well-maintained ground and surface waterworks providing good quality water. The study gives an indication that each DWDS houses a unique eukaryotic community, mainly dependent on the raw water source and water treatment processes in place at the corresponding waterworks. In particular, disinfection as well as hot water temperature seemed to represent a strong selection pressure that controlled the number of active eukaryotic species.

**Electronic supplementary material:**

The online version of this article (10.1186/s40168-019-0715-5) contains supplementary material, which is available to authorized users.

## Background

The water treatment processes, geographical location, pipeline material, raw water type, operational conditions, and water physico-chemical quality shape the microbial communities in drinking water distribution systems (DWDSs) [1–4]. Surprisingly, little information is available about eukaryotic communities in DWDS, impeding sound understanding of the dynamics in DWDS, as co-occurrences such as win-loss interactions (i.e., predator-prey and parasite-host relationships) as well as win-win relationships occur [5]. While diversities of the bacterial and eukaryotic communities have been shown to decline considerably after water treatment processes [6, 7], some members of the eukaryotic community such as amoebae can survive conventional treatments [6, 8, 9].

It is known that some pathogenic amoebae, enteropathogens, and fungi occur in freshwaters [9–11]. Free-living amoebae (FLA) are of specific interest since they can act as human pathogens (e.g., *Acanthamoeba* spp., *Vermamoeba vermiformis*, *Balamuthia mandrillaris*) or serve as hosts for pathogenic microorganisms such as *Legionella* spp. and *Mycobacterium* spp. [12–14]. Fungi in drinking water could be a concern due to the ability of some taxa to produce mycotoxins, although it seems that they are mainly associated with taste or odor problems [15–17]. Invertebrates in aquatic environments may be useful as they can act as predators of microorganisms or contribute to nutrient recycling, especially in water treatment plant filters [6, 18].

The microbial community studies targeting the rRNA genes (DNA) produce taxonomic information including signals from active, dormant, and dead community members. The use of ribosomal rRNA gene transcripts (RNA) has been utilized in previous DWDS studies to differentiate the signals from dead cells and environmental DNA from active and dormant bacterial community members [3, 4]. Further, DNA/RNA library comparisons have been utilized for eukaryotic community characterization in marine [19] and freshwater environments [20]. The objective of this study was to characterize the active or dormant eukaryotic members (analyzed by 18S ribosomal RNA transcript, i.e., rRNA) in comparison with total eukaryotic members (analyzed by 18S ribosomal DNA gene, i.e., rDNA) in DWDS located in different parts of Finland. We aimed to understand the effect of the sampling location, season, and differences between cold water, hot water, and biofilms on the communities within each DWDS. Furthermore, the role of environmental conditions, i.e., geographic location/DWDS, source water type, water treatment process, and disinfection treatment, in shaping the eukaryotic communities, including the occurrence of potentially pathogenic members, was investigated.

## Methods

### DWDS characteristics and sampling

The water samples and biofilm samples were collected from five DWDSs (A–E) from four cities in Eastern and Southern Finland as previously described [21]. DWDSs A–B were located in the same city, and these two waterworks both employed artificial groundwater recharge as a water treatment process. All waterworks were considered as well-maintained and produced good-quality drinking water.

The experimental design for sample collection shown as a chart in Additional file 1 included cold water from three different locations (*N* = 120), hot water from one location (*N* = 40), and water meter biofilms, i.e., soft deposit samples and pipeline biofilms (*N* = 16). The hot water samples originated from warm water systems of buildings heating the drinking water mainly for washing purposes. In each sampling season, water samples in two consecutive weeks per DWDS (A–E) were taken resulting eight water samples per sampling location with the exception of two failed samples of DWDS C (Table 1). Temperatures and total chlorine concentration at each sampling location are described in Table 1. Other measured physico-chemical water quality parameters and concentrations of microbially available nutrients in cold and hot water in each DWDS are summarized in Additional file 2: Tables S1–S2, respectively.

**Table 1:** The sampling locations and characteristics of the samples collected from five DWDSs (A–E). Water samples in each location were collected in four seasons: winter (January to February), spring (March to May), summer (August to September), and autumn (October to December) in two consecutive weeks

DWDS	Water source	Treatment process	Disinfection	Location	Distance (km)	Sample type*	Sample count	Average total chlorine (mg/l)	Range	Average temp. (°C)	Range
A	Artificial groundwater	Aeration, lime stabilization, flocculation, clarification, addition of sulphuric acid, sand filtration	No disinfection	1	2	Cold	8	–	–	12.7	8–18
				2	8	Cold	8	–	–	10.6	7–14
				‘’	‘’	Hot	8	–	–	53.8	51–56
				3	11	Cold	8	–	–	9.2	6–14
				i	na	WM	2	–	–	na	na
B	Artificial groundwater	Aeration, lime stabilization, flocculation, clarification, sand filtration	No disinfection	1	1	Cold	8	–	–	9.6	7–12
				2	3	Cold	8	–	–	10.4	8–13
				‘’	‘’	Hot	8	–	–	57.7	53–62
				3	8	Cold	8	–	–	8.9	6–13
				i	na	WM	2	–	–	na	na
C	Surface water	Ferric sulfate coagulation, flotation, sand filtration, activated carbon filtration	UV-light, ClO_2_, Cl_2_	1	2	Cold	7	0.5	0.4–0.6	8.9	2–20
				2	8	Cold	8	0.3	0.2–0.5	11.8	5–19
				‘’	‘’	Hot	8	na	na	50.6	43–54
				3	20	Cold	7	0.2	0.1–0.4	10.1	6–16
				i	na	WM	2	na	na	na	na
D	Surface water	Ferric sulfate coagulation, clarification, sand filtration, ozonisation, activated carbon filtration	UV-light, NH_2_, Cl_2_	1	5	Cold	8	0.1	< 0.03–0.2	10.3	7–15
				2	14	Cold	8	0.1	0.03–0.4	10.5	6–17
				‘’	‘’	Hot	8	na	na	54.6	51–58
				‘’	‘’	Pipe	3	na	na	na	na
				3	19	Cold	8	0.1	< 0.03–0.3	8.1	5–13
				i	na	WM	1	na	na	na	na
E	Groundwater	Aeration, limestone filtration	UV-light, NaOCl	1	9	Cold	8	0.2	0.1–0.4	6.2	5–9
				2	26	Cold	9	0.1	0.1–0.2	7.0	5–11
				‘’	‘’	Hot	8	na	na	54.1	49–59
				‘’	‘’	Pipe	3	na	na	na	na
				3	36	Cold	8	0.2	0.1–0.5	6.0	4–10
				i	na	WM	3	na	na	na	na

*100 l tap water samples (Cold) and 100 l of water from the warm water system of the building (Hot); water meter biofilm, i.e., loose deposit (WM), pipeline biofilm (Pipe). Pipe includes three replicates from a same pipeline. WM ages (years): DWDS A (14, 15); DWDS B (7, 10); DWDS C (5, 9); DWDS D (8); DWDS E (15, 17, 19). Additional 100 l cold water samples collected with water meter biofilms: *N*(DWDS A) = 2, *N*(DWDS B) = 2, and *N*(DWDS C) = 1. *na* not applicable

Large volume water samples (100 l filtered by a dead-end ultrafiltration method (DEUF); ASAHI Rexeed-25A, Asahi Kasei Medical Co., Ltd., Tokyo, Japan) [22] were collected in the year 2015 with an average flow rate of 3 l/min using a sterile platinum-cured silicone tubing (Masterflex L/S, Cole-Parmer Instrument Co., IL, USA, or Pumpsil, Watson-Marlow Limited, Falmouth, UK) and sterile DIN adapters (Molded Products, Inc., Harlan, USA). Sample volume was measured with a water meter.

Pipe collector biofilm samples; water meter biofilm, i.e., soft deposit samples; water meter-related water samples; and biofilm-related water samples were collected in the late autumn season (Table 1). Pipe collectors (Cross-linked polyethylene (PEX), Uponor) were made of 15 cm pieces with an inside diameter of 0.8 cm. Biofilm from three parallel pieces from the water input side of the pipe collector was removed by shaking 1350 rpm for 3 × 5 min (Heidolph Vibramax, Schwabach, Germany) with sterile 2 mm glass beads (Karl Hecht GmbH & Co. KG, Germany) followed by rinsing with a 5-ml sample water from the same sample point. Biofilm from water meters was detached by brushing and collected using a pipette. In the case that the water meter was empty of water, sterile deionized water was added before brushing. Before further analyses, all biofilm samples were sonicated for 1 min in 40 kHz (Branson Ultrasonics, Danbury, USA).

A 1% solution of sodium thiosulphate (18 mg/ml Na_2_S_2_O_3_·5H_2_O) was added to the water and biofilm samples from chlorinated systems to bind chlorine and prevent residual disinfection after sampling. Before the backflush, excess water was removed by pumping air through a DEUF capsule. Backflush was performed with 500 ml of backflush solution (0.5% Tween 80, 0.01% sodium polyphosphate, and 0.001% Y-30 antifoam emulsion; Sigma-Aldrich and Merck, Darmstadt, Germany) as described in [22] except that a platinum-cured silicon tube (Watson-Marlow Limited, Falmouth, UK) was used to help the collection of DEUF eluate of an average 550 ml (350–580 ml). The secondary concentration of DEUF eluates of an average 75 ml (range 65–145 ml) and concentration of biofilm suspensions of 10 ml was conducted by filtration through a polycarbonate membrane (pore size 0.4 μm, Nuclepore Polycarbonate, Whatman, Kent, UK). Water samples corresponded to the calculated original water volume which was on average of 13.6 l (7.7–25.4 l). The membranes were frozen at − 75 °C or lower before the extraction of nucleic acids.

### DNA and RNA extraction and PCR amplification

Nucleic acids (NAs) were extracted from polycarbonate membranes with Chemagic DNA Plant kit (Perkin Elmer, Waltham, MA, USA) according to the manufacturer’s instructions except that RNA was not removed. Cells were lysed with a buffer and acid-washed 212–300 μm glass beads (Sigma-Aldrich, MO, USA) using a Bead Beater device (BioSpec Products, Inc., Bartlesville, OK, USA) for 1 min at full speed. Nucleic acids were extracted to an elution volume of 100 μl using a Kingfisher device (Thermo Fisher Scientific, Waltham, MA, USA). RNA was purified from 30 μl subsample of NAs with Ambion TURBO DNA-free^TM^ kit (Life Technologies, Carlsbad, CA, USA). Purified RNA was transcribed to complementary DNA (cDNA) using Invitrogen^TM^ Superscript III First-Strand Synthesis System (Thermo Fisher Scientific, Waltham, MA, USA). From all samples, the NAs were used such as the DNA fraction, and the produced cDNA represented the RNA fraction. DNA and RNA concentrations (ng/μl) from extracts were measured with a Qubit minifluorometer using Qubit dsDNA HS Assay and Qubit RNA HS Assay kits (Thermo Fisher Scientific, Waltham, MA, USA). NAs and RNA were stored at − 75 °C and cDNA at − 20 °C prior to sending to LGC Genomics (LGC Genomics GmbH, Berlin, Germany) for further analyses.

Tagged amplicon PCR prior Illumina sequencing was performed using barcoded primers. The PCRs included about 1–10 ng of NAs where the total volume was 1 μl. In addition, 15 pmol of each forward primer and reverse primer was used (in 20 μl: 1× MyTaq buffer containing 1.5 units MyTaq DNA polymerase (Bioline) and 2 μl of BioStabII PCR Enhancer (Sigma)). The eukaryotic primers Eu565F-Eu981R [23] targeting the highly variable V4 region of the 18S rRNA gene were used for PCR amplification (LGC Genomics GmbH, Berlin, Germany). PCR was performed with 30 cycles (including 2 min 96 °C pre-denaturation; 96 °C for 15 s, 50 °C for 30 s, 70 °C for 90 s). PCR amplicon DNA concentrations were checked by gel electrophoresis. Each DNA sample (approx. 20 ng amplicon DNA) was pooled for up to 48 samples carrying different barcodes. If low yields were found in PCR, the corresponding sample was further amplified for 5 cycles. The amplicon pools were purified to remove the primer dimers and other small mispriming products with AMPure XP beads (Agencourt) that were followed by an additional purification on MinElute columns (Qiagen). About 100 ng of each purified amplicon pool was used to construct Illumina libraries using the Ovation Rapid DR Multiplex System 1-96 (NuGEN). Preparative gel electrophoresis was used for Illumina library pooling and size selection. The forward and reverse primers had the same 10 nucleotide barcode sequence in each sample.

### Amplicon sequencing and bioinformatics

Sequencing was performed on an Illumina MiSeq (V3 Chemistry) with 300 bp paired-end reads (Illumina, Inc., San Diego, CA, USA). The libraries were demultiplexed, and barcodes, amplicon primer sequences, and adapter sequences were removed. Reads shorter than 100 bp were discarded. Amplicon reads were processed and analyzed using Quantitative Insights Into Microbial Ecology (QIIME) software [24] version 1.9.1. Quality control for the 18S rRNA gene sequences was performed using Cutadapt [25] to remove adapters. Bad-quality reads were removed with Trimmomatic [26]. The remaining read pairs were joined using Flash2 software [27]. The reads were checked for chimeras with the vsearch algorithm [28]. The reads were clustered at 97% (uclust) into operational taxonomic units (OTUs). The OTU picking step was performed with the open reference OTU picking approach using SILVA SSU database (https://www.arb-silva.de) 128 release [29]. Sequence processing of the samples included negative (*N* = 35) and positive (*N* = 10) controls. From each control, both rDNA and rRNA libraries were analyzed. Negative controls were taken from each sample processing step of water and biofilms and were used to inform the definition of minimum read counts for a sample to be included in the analysis. In-house bacterial and fungal mocks were used as positive controls. One negative filter control (but not samples or other controls) was contaminated with OTU GAZW01284385.3.1621, and this OTU was removed from the dataset prior to analyses. Some of the other negative controls included low read counts of other OTUs (total read count ≤ 2752), and thus, the total read count of the following actual sample (2873) was used as the alpha-rarefaction value. The few OTUs that occurred in negative controls were not removed from the samples due to low amounts in samples.

### Data analyses

The data was analyzed by comparing the representative groups of samples. Alpha-diversity values (Chao1, observed OTUs, Simpson, Shannon) were calculated in QIIME. Beta-diversity values were calculated using binary Jaccard (unweighted) and Bray-Curtis (weighted) metrics. Beta-diversity differences between the samples were displayed by non-metric multidimensional scaling (NMDS) plot in R Version 3.4.2 in the ggplot2 package [30]. Canonical correspondence analysis (CCA) was used to explain the total and active eukaryotic communities and representative taxa by physico-chemical parameters, i.e., temperature; pH; turbidity; absorbance 254 nm; electric conductivity (EC); iron (Fe) concentration; total chlorine; microbially available nutrients, i.e., AOC; MAP; and microbiological water quality parameters, i.e., total cell count (DAPI) and heterotrophic plate count (HPC).

The differences in rDNA and rRNA libraries, i.e., the activity of eukaryotic members, were visualized in a heatmap clustering analysis in the MicrobiomeAnalyst program [31]. First, a combined OTU table was prepared for the samples (*N* = 126) that had successfully produced reads from both rRNA and rDNA libraries. Relative activity recovery (%) for each OTU was determined by subtracting the relative abundance of DNA from the relative abundance of RNA (RNA% − DNA%). Recovery values ranged from − 100 to 100% in which the taxon was considered active if the rRNA relative recovery was greater than its recovery from rDNA [20]. The relative recovery OTU table at rank D4 to D6 level was filtered by variance (standard deviation filtering 70%) to select only the OTUs (*N* = 521 OTUs) that displayed the most variation in activity recovery and between the samples of the entire dataset. In addition, clustering analysis was performed separately for the most abundant kingdoms using the entire dataset without filtering to capture all OTUs. The figures of the heatmap clustering analysis included scaling by rows, i.e., per taxon in the entire dataset, clustering was based on a default Euclidean distance and Ward clustering methods.

### Statistical analysis

Analysis of similarity (ANOSIM) for NMDS plots was performed in QIIME and in MicrobiomeAnalyst. In QIIME, the script compare.categories.py was used for analyzing the differences in the entire datasets between the DWDSs (Bray-Jaccard and Bray-Curtis). Within each DWDS, the analysis of beta-diversity (Bray-Curtis) was conducted for DNA and RNA separately to investigate the (1) differences between RNA/DNA in each DWDS (all samples), (2) effect of season and location only from cold water samples, and (3) differences between cold and hot water (location 2 only). IBM SPSS Statistics 25 (IBM Corporation, USA) was used for the statistical analyses of alpha-diversities. The effect of season and sampling location to alpha-diversities was tested using non-parametric Kruskal-Wallis test or Mann-Whitney *U* test (RNA/DNA) within each DWDS and all DWDSs in cold water (four seasons, three sampling locations). The effects of sample type to alpha-diversities in the entire dataset and within DWDS were tested with the Kruskal-Wallis test. Significance level *P* = 0.05 was used in all statistical analyses.

## Results

### Eukaryotic community characterization and differences between DWDSs

Overall, the 140 rDNA and 146 rRNA libraries representing total and active eukaryotic communities, respectively, resulted in 2.6 rDNA and 4.0 million rRNA amplicon reads (Additional file 3: Table S3). A total of 40 rDNA and 34 rRNA samples originating mostly from DWDS E (both cold and hot water) produced libraries with low read counts, which were excluded from the analysis.

**Fig. 1:**
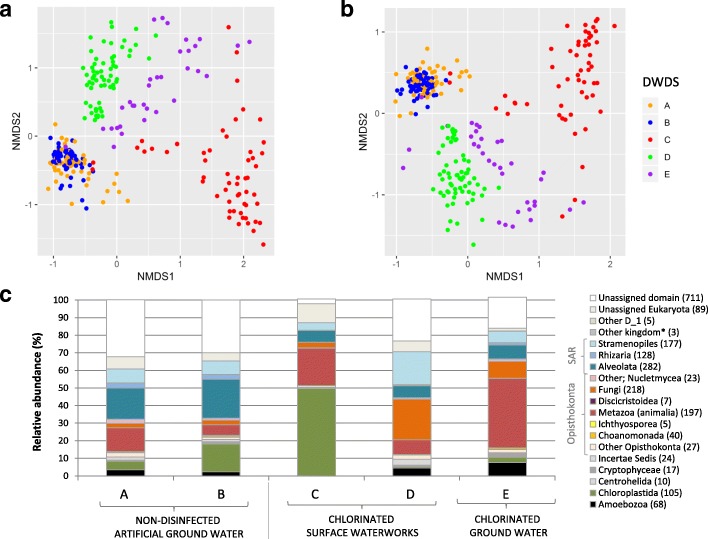
Eukaryotic community characteristics of the drinking water distribution system samples. NMDS plot of **a** unweighted binary Jaccard (ANOSIM *r* = 0.73, *P* < 0.001) and **b** weighted Bray-Curtis (ANOSIM *r* = 0.73, *P* < 0.001) beta-diversities and **c** representative kingdoms within DWDSs A–E. Number of OTUs within each kingdom in brackets, *Discoba, *Malawimonas*, *Haptophyta*

The samples were clustered for DWDSs A–E. All samples originating from DWDSs A and B clustered close to each other in both unweighted and weighted non-metric multidimensional scaling plots (NMDS) (Fig. 1a, b), i.e., the beta-diversity of eukaryotic communities at non-disinfected DWDS samples was similar. DWDSs C–E clustered more clearly apart from each other in the NMDS plots, reflecting a certain level of uniqueness of all disinfected DWDSs. This was evident also in the differences in the relative abundance of the eukaryotic kingdoms in the different DWDSs (Fig. 1c). *Alveolata* dominated in DWDSs A and B, *Chloroplastida* in DWDS C, *Fungi* in DWDS D, and *Metazoa* in DWDS E. A high abundance (20–41%) of unassigned OTUs was present in all DWDSs resulting in a total of 711 OTUs (Fig. 1c). The entire eukaryotic dataset consisted of 2136 OTUs that distributed into taxonomic groups mainly in seven kingdoms as presented in Table 2.

**Table 2:** The number of OTUs in the most abundant taxonomic groups identified from the DWDSs

Kingdom	Subkingdom/Phyla/Clade	Number of OTUs
*Metazoa*	*Arthropoda* (mainly *Copepoda*, small crustaceans, i.e., copepods)	48
	*Gastrotricha* (i.e., hairy back worms)	13
	*Nematoda* (roundworms, i.e., nematodes)	63
	*Rotifera* (wheel animalcules, i.e., rotifers)	35
	*Platyhelminthes* (flatworms, i.e., platyhelminths)	12
*Fungi*	*Ascomycota*	66
	*Basidiomycota*	68
	*Cryptomycota*	53
*Chloroplastida*	*Charophyta*, including land plants *Embryophyta*	68
	*Chlorophyta*, including green algae *Trebouxiphyceae*	36
*Amoebozoa*	*Discosea*	24
	*Tubulinea*	22
	*LEMD255*	7
	*LKM74*	12
*Alveolata*	*Ciliophora*, containing hair-like cilia, i.e., ciliates	225
	*Dinoflagellata*, phytoplankton, i.e., dinoflagellates	42
	*Protalveolata*	17
*Stramenopiles*	*Ochrophyta*, golden-brown algae	174
	*Peronosporomycetes*, water mould, i.e., oomycota	19
*Rhizaria*	*Cercozoa*, single cell flagellates	127

**Fig. 2:**
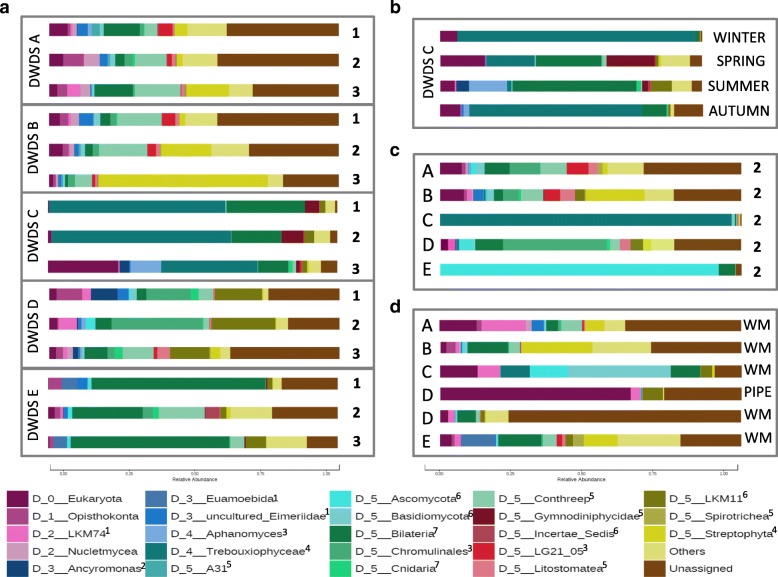
Average relative abundances of the most abundant active (RNA) eukaryotic taxa characterized to D5 taxa level. **a** DWDSs A–E: cold waters at locations 1–3. **b** DWDS C: cold waters between seasons. **c** DWDSs A–E: hot waters at location 2. **d** DWDSs A–E: biofilms from water meters (WM) or pipelines (PIPE). Taxa “others” represent OTUs that contain < 3000 reads in the entire dataset. Kingdoms marked as upper index: ^1^*Amoebozoa*, ^2^*Incertae Sedis*, ^3^*Stramenopiles*, ^4^*Chloroplastida*, ^5^*Alveolata*, ^6^*Fungi*, ^7^*Metazoa* (Animalia)

An analysis of eukaryotic taxa characterized to rank level D5 confirmed similar taxa profiles in DWDSs A and B, while DWDSs C–E each displayed unique taxa profiles in both RNA (Fig. 2) and DNA analyses (Additional file 4: Figure S1). In DWDSs A and B, the high occurrence of *Alveolata* consisted of ciliate class *Intramacronucleata* (order *Conthreep*) and *Perkinsidae* class (order A31). In DWDS C, green algae *Trebouxiophyceae* class was mostly responsible for the high abundance of *Chloroplastida*. In DWDS D, the high abundance of *Fungi* consisted mainly of subphylum LKM11 of *Cryptomycota* phylum, and there was also a high abundance of golden-brown algae order *Chromulinales* (*Ochrophyta*). The abundant *Metazoa* occurrence in DWDS E included nematodes but also rotifers and copepods. A more detailed analysis of the most abundant top 50 OTUs in each DWDS is presented in Additional file 5: Tables S4–S8.

**Fig. 3:**
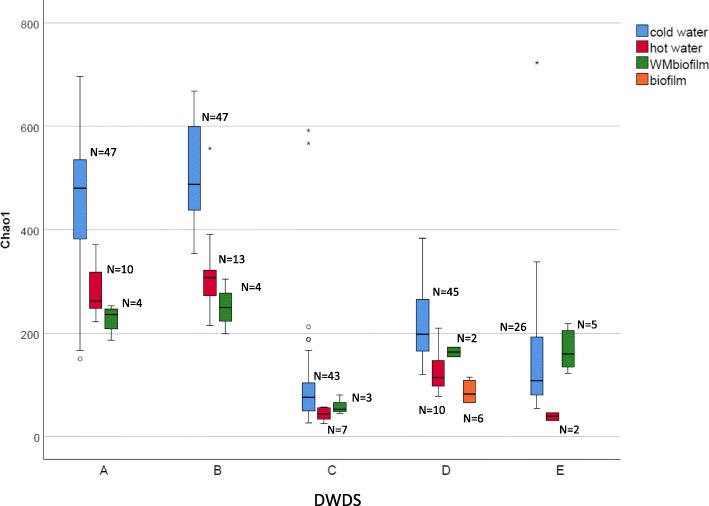
Boxplot of alpha-diversity Chao1 values grouped by cold water, hot water, and biofilms in each DWDSs A–E (DNA + RNA). WMbiofilm, water meter biofilm; biofilm, pipeline biofilm; *N*, number of samples

Alpha- and beta-diversities revealed the differences in DWDSs A–B when compared to DWDSs C–E, most likely due to disinfection. Species richness as estimated via Chao1 (Fig. 3) and Shannon alpha-diversity estimator (Additional file 6: Figure S2) was clearly higher in non-disinfected samples from DWDSs A–B as compared to disinfected samples from DWDSs C–E.

### Eukaryotic communities in cold and hot water, as well as in biofilms

Alpha-diversity differed significantly between the samples of cold water, hot water, and biofilms (Chao1, *P* < 0.05, Fig. 3; Shannon index, *P* < 0.05, Additional file 6: Figure S2), with the highest values occurring in cold water samples. The differences were more pronounced in non-disinfected DWDSs A–B and disinfected DWDS D (Chao 1, *P* < 0.001) as compared to disinfected DWDSs C and E (Chao1, *P* < 0.05).

**Fig. 4:**
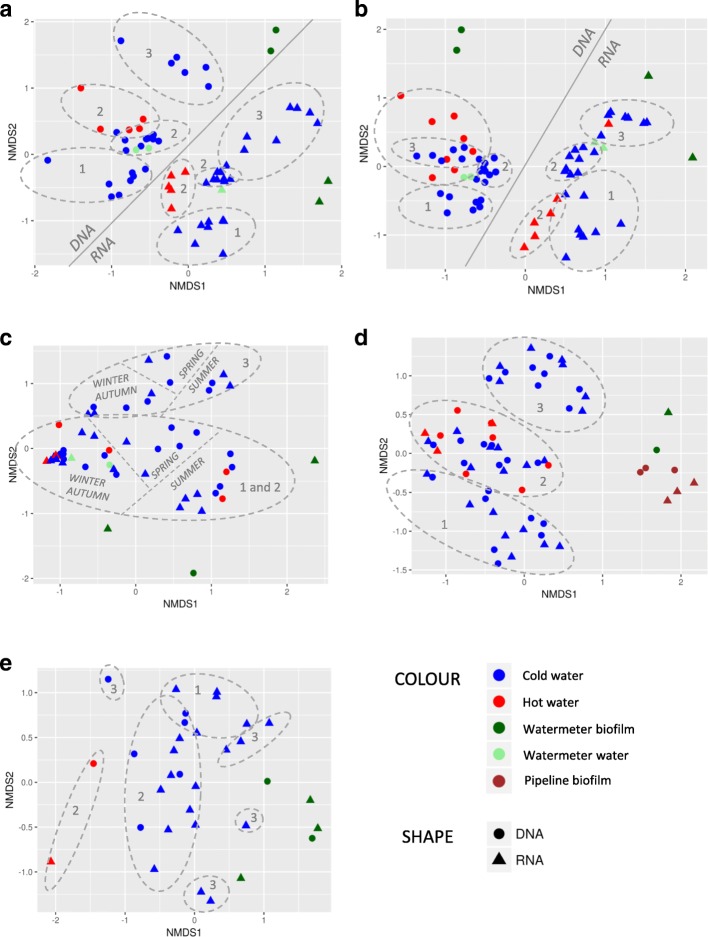
**a**–**e** NDMS plots of weighted Bray-Curtis beta-diversity within each DWDSs A–E. Water samples at different locations 1–3 marked as dashed circles; RNA/DNA and season marked if clear separations occur

The richness of eukaryotic species (Chao1) was significantly lower in hot water than in cold water (Fig. 3; Kruskal-Wallis pairwise comparison, *P* < 0.05) while Shannon diversity was not (Additional file 6: Figure S2). In detailed NMDS plots highlighting the differences between the sample types of DWDSs A and B, clusters according to beta-diversity in both DNA and RNA were obvious (*R* = 0.47–0.70, *P* < 0.01, ANOSIM) (Fig. 4a, b) and hot water samples clustered away from the corresponding cold water samples (sampling location 2) in both RNA (*R* > 0.7, *P* < 0.01) and DNA (*R* > 0.5, *P* < 0.002). When targeting RNA, cold water differed from hot water also in DWDS D (*R* ≥ 0.45, *P* < 0.05). From the most abundant taxa profiles, hot water included the same taxa as the corresponding cold water, but slightly different proportions in DWDSs A–D (Fig. 2, Additional file 4: Figure S1). In DWDS D, the relative abundance of active fungi LKM11 in hot water (4%) was lower than that in cold water (14–22%) (Fig. 2). In DWDS E, hot water samples differed from cold water and showed an especially high abundance of fungi *Ascomycota* (92% RNA, 25% DNA).

Water meter biofilms showed lower alpha-diversity values than cold water only in unchlorinated DWDSs A and B (Fig. 3, Additional file 6: Figure S2). Biofilm samples clustered away from water samples in the weighted beta-diversity NMDS plots in all DWDSs A–E (Fig. 4). Biofilms displayed a difference in the most abundant taxa profiles when compared to water samples from the same DWDS (Fig. 2, Additional file 4: Figure S1). The most abundant taxa in biofilms varied between DWDS but *Bilateria* (nematodes, copepods, rotifers) and fungal subphylum LKM11 were abundant in RNA (Fig. 2) and DNA samples (Additional file 4: Figure S1). In biofilm RNA samples, there was also an abundance of the amoeba clade LKM74, *Streptophyta*, *Conthreep* (ciliates), and *Chromulinales* (golden-brown algae) (Fig. 2).

### Total and active eukaryotic communities in cold water from different DWDS locations and seasons

**Fig. 5:**
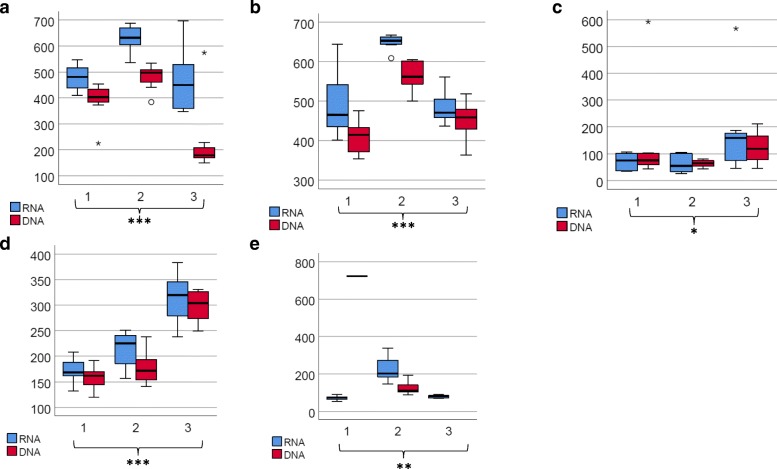
**a**–**e** Boxplot of alpha-diversity Chao1 values in cold water within DWDSs A–E. Values are separated by location (1 to 3) and by template RNA/DNA in the drinking water distribution system. Statistical significance of the location (RNA + DNA): *P < 0.05; **P < 0.01; ***P < 0.001

The effect of location inside the DWDS was evident as differences in alpha- and beta-diversities and in the increased abundance of certain taxa. In DWDSs A–B and D–E, beta-diversities were clearly location-dependent according to both DNA and RNA (ANOSIM, *R* = 0.6–0.8, *P* ≤ 0.01), but this was less evident in DWDS C (*R* = 0.2–0.3, *P* ≤ 0.02) (Fig. 4). Statistical analysis was not conducted in DNA in DWDS E due to the low sample counts. Furthermore, also Chao1 and Shannon alpha-diversity indices were significantly different between the locations in all DWDSs except in DWDS C (Fig. 5; Additional file 6: Figure S3). Chao1 index in non-disinfected DWDSs A–B (Fig. 5a, b) and Shannon index in DWDS A (Additional file 6: Figure S3) differed between RNA and DNA (*P* < 0.05). Different locations mostly shared the same total and active common taxa within each DWDS but with different relative proportions (Fig. 2, Additional file 4: Figure S1).

The season exerted significant effects on the eukaryotic community in DWDS C only. This temporal effect on beta-diversity (RNA and DNA: *R* = 0.6–0.7, *P* = 0.01) was even more pronounced than the spatial effect (Fig. 4c). Higher species richness and larger diversity values were detected in spring and summer (Chao1 60–592, Shannon 1.7–2.4) compared to winter and autumn seasons (Chao1 26–159, Shannon 1.0–3.5). In samples with low species richness from winter and autumn, we observed a high coverage of one taxon, green algae *Trebouxiophyceae* (Fig. 2b, Additional file 4: Figure S1). No such seasonal differences were detected in the other DWDSs.

### Active and non-active eukaryotic members in DWDS

**Fig. 6:**
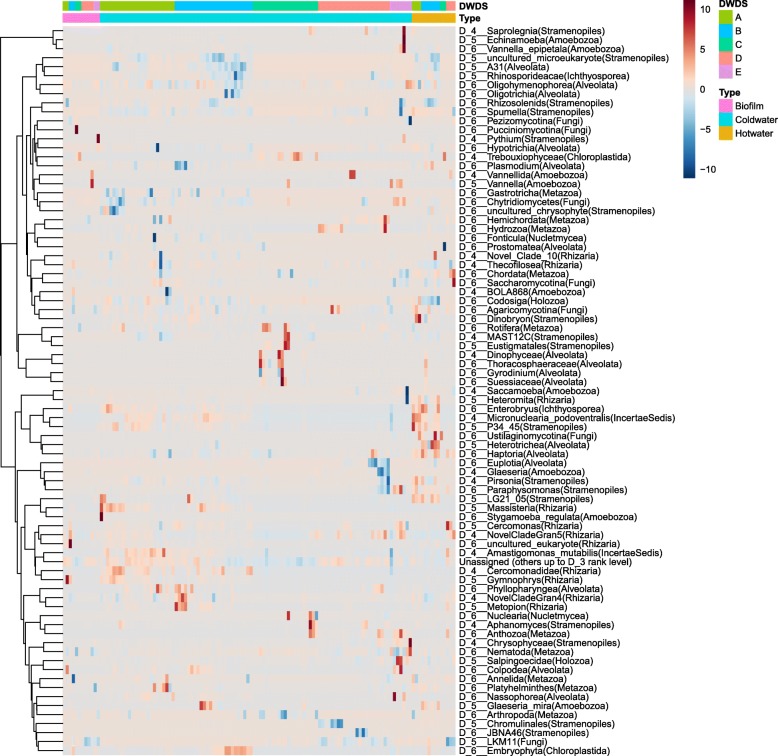
Heatmap clustering analysis of eukaryotic members by relative activity recovery (%). Only the most important OTUs (30% highest variation in the dataset) and their representative taxa ranked from D4 to D6 level are shown (see the entire dataset by kingdoms in Additional file 7: Figures S4–S10). Red color represents a high activity recovery, i.e., relative abundance (%) is higher in the RNA than the DNA library, and blue color represents low activity recovery in the sample. Kingdom marked inside the brackets

Clustering analysis suggested some active and non-active OTUs as determined via the calculation of activity recovery, i.e., RNA% − DNA% (Fig. 6). Some taxa clustered according to the differences in activity between cold water, hot water, and biofilms, or between the DWDSs, while many representative taxa showed random activity recovery. The most abundant kingdoms seemed to contain both active and non-active taxa (Additional file 7: Figures S4–S10). However, some kingdoms such as *Chloroplastida* and *Amoebozoa* showed higher activity recovery than other kingdoms (Additional file 7: Figures S4 and S7). On the contrary, many *Fungi* including LKM11 that represented the most abundant fungal taxon appeared to be inactive (Additional file 7: Figure S5).

Some ciliates (multiple *Alveolata* taxa) seemed active only in non-disinfected DWDSs (Additional file 7: Figure S8). In DWDS C, phytoplankton *Dinoflagellata* (e.g., *Gyrodinium*, *Biecheleria*, *Suessiaceae*) (Additional file 6: Figure S8) showed increased activity recovery in spring and summer whereas green algae *Chlorophyta* (e.g., *Trebouxiphyceae*) seemed to be active in the winter and autumn seasons (Additional file 7: Figure S7). There were few *Stramenopiles* taxa of the golden-brown algae *Ochrophyta* in DWDSs A–B (Additional file 7: Figure S9) as well as ciliate taxa (Additional file 7: Figure S10) in DWDSs B and D which seemed to be active in hot but not in cold water.

### Potentially pathogenic eukaryotic members

Few genera of free-living amoebae were found in cold water, hot water, or biofilms at low read abundance (Additional file 8: Tables S9–S11). The potentially pathogenic *Balamuthia* were found mainly in cold water originating from non-disinfected water from DWDSs A–B (prevalence 17–88% of the samples). Very low read counts of potentially pathogenic amoeba *Vermamoeba vermiformis* were found in cold and hot water in DWDSs A–D and especially from the pipeline biofilms from DWDS D (Additional file 8: Tables S9–S11). Six OTUs of free-living amoeba *Vannella* were present in the dataset, the highest abundance occurred in cold waters from DWDS E (*Vannella epitetala*) (Additional file 8: Table S9). Furthermore, amoebas *Vannella* and *Vermamoeba* seemed to be active according to the activity recovery calculation (Additional file 7: Figure S4). A few fungal genera that might cause adverse health effects were found: *Aspergillus*, *Candida*, *Paecilomyces*, *Stachybotrys*, *Alternaria*, and *Penicillium*, with the latter showing the highest abundances, especially in cold water (Additional file 8: Tables S9–S11). Some of these fungi, e.g., *Penicillium* and *Candida*, did not seem to be active in any of the samples (Additional file 7: Figure S5).

### Effects of environmental factors for eukaryotic communities in cold water

**Fig. 7:**
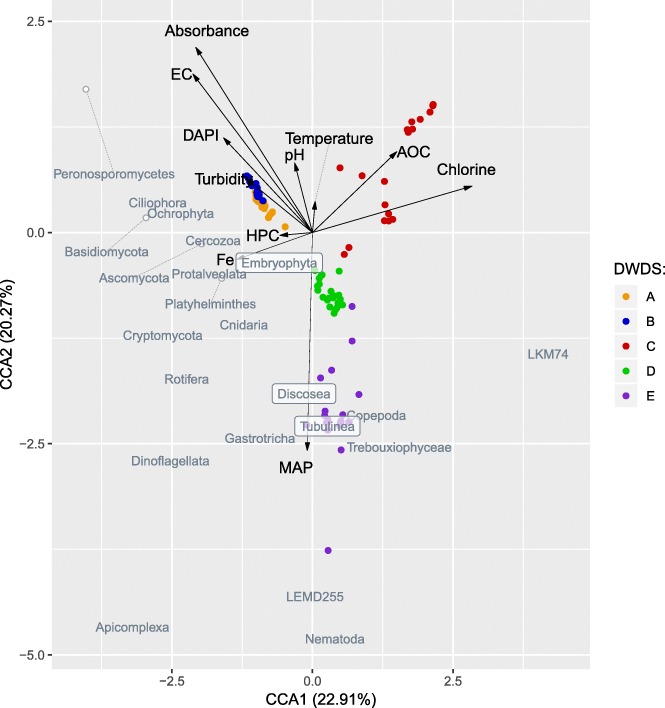
Active (RNA) eukaryotic community and representative taxa in association with physico-chemical and microbiological parameters of the water. Explanation rates for CCA1 and CCA2 axes in canonical correspondence analysis (CCA) are in the brackets. Each point represents a eukaryotic community of one sample colored according to the DWDS, and gray text indicates the most abundant eukaryotic taxa (overlapping taxa marked with white circles (exact location) or transparent boxes for clarification). Representative taxa per kingdom: *Amoebozoa* (LKM74, LEMD255 and *Discosea*, *Tubulinea* phyla), *Alveolata* (*Dinoflagellata*, *Ciliophora*, *Apicomplexa* phyla), *Fungi* (*Ascomycota*, *Basidiomycota*, *Cryptomycota* phyla), *Rhizaria* (*Cercozoa*), *Metazoa* (*Nematoda*, *Copepoda*, *Cnidaria*, *Rotifera*, *Platyhelminthes* phyla), *Chloroplastida* (*Embryophyta*, *Trebouxiphyceae* class), and *Stramenopiles* (*Peronosporomycetes*, *Ochrophyta* phyla). Scaling and other details of the CCA are described in conjunction with DNA-based eukaryotic community analysis, Additional file 9

The canonical correspondence analyses for active (RNA; Fig. 7) and total (DNA; Additional file 9: Figure S11) eukaryotic communities and representative taxa indicated that chlorine seemed to be an especially important factor shaping the composition of the eukaryotic community. High chlorine concentrations were found to be inversely correlated with the abundances of the most abundant phyla and classes from all of the major kingdoms (*Amoebozoa*, *Fungi*, *Alveolata*, *Metazoa*, *Chloroplastida*, *Stramenopiles*, *Rhizaria*). The effect was more clearly seen in RNA analyses, evidence that chlorine exerted an effective disinfection property against the active eukaryote population. The samples from DWDS C that on average contained higher total chlorine concentrations (0.3 mg/l) than DWDSs D and E (0.1 and 0.2 mg/l, respectively) (Table 1) clustered in the correspondence analysis. In addition to chlorine, the high assimilable organic carbon (AOC) concentration seemed to shape the active and total eukaryotic communities, especially the presence of *Trebouxiphyceae* in the DNA library of DWDS C (Fig. 7, Additional file 9: Figure S11). The concentration of microbially available phosphorus (MAP) in water correlated with the presence of active eukaryotic community in DWDSs D and E (Fig. 7). DNA and RNA samples from DWDSs A and B clustered along the increased values of water absorbance at 254 nm, electric conductivity, turbidity, and total cell counts (Fig. 7, Additional file 9: Figure S11). In general, water temperature and heterotrophic plate count (HPC) exerted only minor effects on the eukaryotic communities in the entire dataset.

## Discussion

### The waterworks itself is an important environmental driver shaping eukaryotic communities

By utilizing the amplicon sequencing method, we were able to characterize a diverse group of eukaryotic inhabitants of drinking water and biofilms both with respect to total eukaryotic organisms (DNA) and to those which were considered as active (RNA). Our analysis assayed the 18S rRNA genes and the respective transcripts originating from five different and well-functioning large-scale waterworks and detected kingdoms that are commonly reported in DWDS, i.e., *Ameobozoa*, *Alveolata*, *Metazoa*, *Chloroplastida*, *Fungi*, *Stramenopiles*, and *Rhizaria* [6–8, 32–41]. This study showed that the eukaryotic communities in DWDS were dependent on the origin of the raw water, the geographical location of the waterworks, and the water treatment process.

The importance of waterworks has been previously reported with respect to the formation of bacterial communities [1, 42]. Treated drinking water may resemble raw water when some organisms are able to survive the treatment process [34]. In our study, DWDSs A and B were located in the same city, used artificially recharged groundwater, did not apply disinfection, and shared a similar structure of the eukaryotic community. One could speculate that the combination of a distinctive groundwater formation and the lack of treatment in waterworks A and B together resulted in the higher particle or ion concentrations (turbidity, absorbance 254 nm, electric conductivity) and total cell counts; these were observed to be important environmental drivers in CCA and further were connected to rich and diverse eukaryotic communities. However, in general level, the most common eukaryotic members such as phytoplankton, ciliates, amoebas, fungi, and invertebrates were present in all studied DWDSs.

In this study, the eukaryotic communities had been most likely affected by the different water treatment processes in addition to the differences in raw water sources. The water treatment process is known to influence important microbial taxa such as free-living amoeba [40, 43], fungi [11], and the entire eukaryotic community [6]. Filtration in the water treatment process is an important stage in the elimination of microbes such as FLA and protozoan (oo)cysts [9]. On the other hand, a variety of diverse eukaryotic communities containing invertebrates (rotifers, nematodes, gastrotrichs), *Fungi*, and *Cercozoa* can colonize both the sand filtration and the active carbon filtration processes [15, 18, 44–47]. Despite the spatial effects in DWDS, the eukaryotic communities in water seem to reflect the shifts in the eukaryotic communities occurring in the water treatment processes as it is possible for microorganisms to become detached from the filters into the final treated drinking water [45]. The complex food web in some filters may promote growth, and therefore, the backwashing of filters is especially important for the control of eukaryotic community [6, 40, 44, 47]. In some cases, clogged filters may explain the especially high occurrence of some eukaryotes also found in this study such as nematodes and microcrustaceans [18].

Disinfection by UV irradiation and chlorine as the last step of the water treatment process is a powerful means to reduce the growth of the bacterial species in DWDSs [6]. In this study, significantly higher eukaryotic species richness occurred in unchlorinated DWDSs A–B in comparison with chlorinated DWDSs C–E, suggesting that chlorination has efficacy also in decreasing active eukaryotic populations. This selective pressure caused by disinfection was seen as a more homogeneous eukaryotic population, in other words, a less diverse community, a phenomenon earlier found to occur in bacterial populations [4]. In addition, differences in alpha- and beta-diversities were observed between total and active eukaryotic communities only in non-disinfected DWDS, suggesting that more eukaryotic taxa were active in unchlorinated DWDS. The high relative abundances of some active taxa in disinfected DWDS suggest these taxa can tolerate chlorine to some extent. Especially in DWDS C, i.e., the waterworks which utilized the highest chlorine concentrations (average 0.3 versus 0.1 and 0.2 mg/l in DWDSs D and E, respectively), phytoplankton (dinoflagellates, green algae *Trebouxiphyceae* and water mould *Spumella*), and *Metazoa* (copepods, rotifers, and nematodes) dominated. These eukaryotes have been found in DWDS utilizing even higher chlorine concentrations than examined here [39]. For example, *Monogononta* from the rotifer class, a microorganism commonly found in chlorinated DWDS, are reported to produce dormant eggs that are resistant to environmental conditions [48].

### Eukaryotic communities in biofilms and hot water display different characteristics to those in cold water

Previously, biofilms have been reported to have different eukaryotic communities than the water in the DWDS [6]. In this study, the biofilms seemed to be more uniquely representative of the water system in which they were living rather than bearing similarities to each other. The biofilms sampled from all DWDSs showed less eukaryotic species richness and diversity than the corresponding water samples, suggesting that only some of the eukaryotic members can thrive in biofilms, such as *Amoebozoa* [38]. These biofilms, however, did not seem to be particularly selective for certain kingdoms as all major kingdoms also present in water were detected in biofilms (*Cloroplastida*, *Metazoa*, *Alveolata*, *Stramenopiles*, *Amoebozoa*). In case of pathogenic members, the biofilm detachment may pose a health risk to water consumers. In this study, the occurrence of *Vermamoeba vermiformis* amoeba in DWDS D biofilms is of special interest despite its low abundance (≤ 50 reads/biofilm sample).

A hot water temperature is known to decrease the active microbial biomass and change the characteristics of the bacterial community and its metabolic functions [2]. In our present study, it was found that a hot water system represented a selective pressure also for eukaryotes decreasing the number of the rare eukaryotic species in hot water. Interestingly, in all DWDSs, the most abundant eukaryotic taxa were shared between the cold and hot water with relative similar abundances (mainly *Bilateria*, *Streptophyta*, *Chromulinales*, *Conthreep*, green algae *Trebouxiphyceae*). These taxa were clearly robust and tolerated hot water temperatures. In particular, a water mould, genus *Spumella*, seemed to benefit from the changed environmental conditions that led to its increased relative abundance either due to its growth or relatively higher resistance to hot water. Not all of the abundant eukaryotic organisms were able to survive in such harsh conditions. For example, the most abundant fungi found in this study, *Cryptomycota* (LKM11), showed generally low activity recovery and a lower abundance in hot water than in cold water.

### Different locations within a DWDS represent distinct communities while the effects of season are visible under some circumstances

There were invariable differences detected in the eukaryotic communities between different locations within the full-scale DWDSs studied herein, with this being mainly evident in species richness and differences in beta-diversity. Only in DWDS D did the number of eukaryotic species increases with growing distance from the waterworks. In DWDS C, the different alpha- and beta-diversities in the most distant location 3 compared to locations 1 and 2 may be due to the lower residual chlorine concentration or higher iron concentrations in the site [21]. In DWDS B, an increased abundance of active *Tarenaya hassleriana* was detected as the distance from the water treatment plant increased. The reason is unclear, but it might be due to its growth in DWDS, detachment from biofilms, or increased RNA due to the adaptation to stress conditions such as increased darkness [49]. The findings are in accordance with earlier reports indicating that at DWDS endpoints, one can detect an increase in the occurrence and diversity of eukaryotes, e.g., amoebas [8].

Only in DWDS C was the eukaryotic community in the water affected by season, with winter and autumn samples resembling each other [39]. The pronounced effect of season was particularly evident in the occurrence of phytoplankton. A green algae *Trebouxiophyceae* dominated winter and autumn samples whereas the activity recovery and occurrence of dinoflagellates were elevated in the samples collected in spring and summer. The quality of raw water source (surface water) may be reflected in the treated water, especially if obligate photoautotrophic microorganisms are present and the algae can survive the water treatment process [39]. Growth within the DWDS is also a feasible explanation as the green algae *Trebouxiphyceae* class contains non-photosynthetic species that could thrive in dark DWDS environments [50]. The high relative abundance of phytoplankton might be also due to the reduction in the numbers of other eukaryotic members which was observed as low species richness and less diversity in winter and autumn seasons. The higher relative abundance of *Bilateria* invertebrates (copepods, nematodes, and rotifers) observed in spring and summer [39] might be due to their bacterivorous properties, i.e., these benefit from the increased counts of heterotrophic bacteria that occurred especially in summer season [21]. In DWDS of the other surface waterworks (DWDS D), the effect of season was not obvious, and thus, the source water type alone cannot explain the observed seasonal differences in DWDS C.

### Many eukaryotic taxa show differences in their activity recovery depending on environmental conditions

This novel study characterized the eukaryotic communities in drinking water utilizing also RNA as a template. Activity recovery (RNA–DNA) [20] was considered a more appropriate way to handle this data instead of the RNA/DNA ratio [19, 51, 52] in order to keep also zero values in the analysis. In the relative activity recovery, the scale was limited to lower variation (− 100 to 100), and thus, the distribution of the results was even more extensive than in the RNA/DNA ratio calculation (scale in this data was − 3400- to 14,600-fold).

Heatmap clustering analysis suggested increased activities based on the higher relative abundance in RNA than DNA samples of some eukaryotic taxa under certain circumstances. *Amoebozoa* and *Chloroplastida* seemed active in most DWDS whereas other kingdoms seemed to exhibit more variability and contain both active and non-active taxa. Of phytoplankton taxa, dinoflagellates seemed to be active in some DWDS in the spring and summer seasons as well as green algae *Trebouxiphyceae* (DWDS C) and spider flower *Tarenaya hassleriana* (DWDS B). Further, algal phylum *Ochrophyta* members were active in hot water. On the other hand, fungal subphylum LKM11 and ciliates showed low activity recovery, most probably due to chlorination.

While delivering novel information, also the limitations in the interpretation of activity recovery results solely as a sign of increased activity must be noted. A phenomenon where an increased ribosomal RNA count occurs after the bacterial cells become dormant [49, 53] is suspected also to occur in eukaryotes during environmental stress events such as increased chlorination, elevated temperature, or lack of nutrients. On the community level, the species richness is expected to be higher in rDNA libraries compared to rRNA libraries as rRNA only describes active or dormant population but excludes environmental DNA originating from dead cells [3]. However, due to the higher rRNA than rDNA copy counts in a cell, the RNA approach may better detect rare taxa as previously noted as higher species richness in RNA than in DNA sequence libraries of bacterial communities in drinking water [4]. In the present study with eukaryotes, higher species richness in RNA than in DNA samples was noted especially in unchlorinated cold water, the finding potentially indicating the more sensitive detection of rare community members when targeting sequencing efforts to rRNA instead of rDNA. The variation in the ribosomal locus may be more extensive in genomes of eukaryotes (extending to tens of thousands of copies) than in bacteria (some dozens) [54] affecting the detection sensitivity.

### Evaluation of some opportunistic pathogens or harmful eukaryotic members

In the studied DWDS, the finding of six different OTUs of *Vannella* spp. is a concern, due to the ability of certain *Vannella* spp. to host pathogenic amoeba-resisting bacteria such as *Legionella* [55]. In addition, low reads of potentially pathogenic amoebal OTUs were identified as *Balamuthia* (maximum 42 reads/sample in water) and *Vermamoeba vermiformis* (≤ 50 reads/sample in biofilms). In addition to amoebas, *Paramecium* and *Cyclidium* ciliates were found and these are capable to act as hosts for pathogens, e.g., *Legionella* [56]. Also, a few OTUs of the *Hymenostomatia* subfamily were found, but these could not be identified to a deeper level; however, this taxon contains *Tetrahymena* genera known to act as a host for pathogens [56].

The relative abundances of potential pathogens in this study were low in comparison with earlier reports from other DWDS including reservoirs where *Acanthamoeba* and *Vermamoeba* can be present even as major amoebal members [8, 38]. While FLA risks are still poorly identified, *Vannella* species and *V. vermiformis* have been detected in treated drinking water in the UK, Canada, and the USA [57]. Although the members of these taxa are rarely linked with human disease, they have the potential to cause severe infection in humans and may also pose indirect public health issues by hosting pathogenic bacteria [12–14, 58, 59]. A more detailed identification will be needed to confirm the presence of pathogenic genera, as the OTUs detected represented new reference OTUs. The sources of rare *Balamuthia* brain infections in the USA have been linked in addition to the soil environment also to different water sources [59]. One case of *Balamuthia* was linked to nasal lavage with drinking water containing this amoeba [60].

Similar to our findings, many researchers have found fungal genera *Penicillium*, *Aspergillus* [6], *Alternaria*, and *Candida* [39] in the DWDS, and some species of these genera can cause negative health outcomes [16, 61, 62]. In addition, fungi cause esthetic problems in DWDS [15]. Also, nematodes cause esthetic concerns but may additionally carry pathogenic bacteria in their gut, thus representing a potential health hazard [37, 63]. However, the common abundance of nematodes in DWDS [37] combined with the lack of reported health problems suggests that these are a normal part of the DWDS biota, and their presence may even be beneficial in water treatment filtration processes [18, 46, 64].

### Taxonomic identification limitations

Universal eukaryotic primers are designed to target a wide range of eukaryotes. In this study, the primer pair Eu565F-Eu981R [23] was used for the first time in a DWDS study and revealed a wide eukaryotic community containing all of the relevant DWDS kingdoms. However, it was not possible to identify many abundant taxa from important kingdoms such as *Fungi* and *Amoebozoa* to a deeper taxonomic identification level in the SILVA database. This suggests that not all eukaryotic taxa members that are abundant in nature are represented in this database. For *Fungi*, a high abundance of LKM11 subphylum of *Cryptomycota* was observed. It is only recently that this uncultivable group of fungi has been detected in environmental freshwaters [65]. The possible ecological importance of this “hidden fungi” in DWDS should be clarified; in fact, future research needs have been already acknowledged in a study on freshwater environments [66], including the ecological and health-related aspects of this fungi. For *Amoebozoa*, the most abundant taxa were LKM74 [8] and LKM255 clades whose ecological role is still unresolved; the LKM74 clade is a common soil and freshwater inhabitant, but it has been possible to cultivate only one species, *Mycamoeba gemmipara* [67], highlighting non-cultivability and novelty of the members in LKM74 clade. Furthermore, traditional methods as well as more taxa-specific primers, e.g., the fungi-specific primer internal transcribed spacer (ITS), are still useful for identification purposes. In addition, there is an obvious need to expand the reference databases, e.g., SILVA for better characterization of eukaryotic organisms [11, 68].

## Conclusions

This study examining DWDSs of well-functioning waterworks revealed that diverse eukaryotic communities (invertebrates, i.e., nematodes, rotifers, and ciliates; fungi; phytoplankton; and amoebas) exist in DWDS as part of the normal biota.

The eukaryotic communities present in the DWDS were most strongly shaped by the waterworks rather than season, the location within the DWDS or the water temperature. This highlights the independent or combined role of the raw water source, geographical location, and/or water treatment process in determining which taxa will be present.

The lower number of eukaryotic species in the disinfected DWDSs and in hot water as compared to non-disinfected cold water indicates that these circumstances exert selective pressure on which eukaryotic members in the DWDS can survive. *Metazoa*, *Fungi*, and *Chloroplastida* appeared to be the most resistant eukaryotic members as judged by their high abundances in disinfected systems.

The ecological role and risk assessment of some of the common eukaryotic inhabitants of the DWDS found here, such as free-living amoebae, fungi, and invertebrates like roundworms *Nematoda*, should stimulate future research. Moreover, a deeper taxonomic characterization of the abundant but still unidentified taxa, especially within the *Amoebozoa* and *Fungi* kingdoms, should be recognized.

## Additional files

Additional file 1: A chart of the experimental design of the study. (PDF 100 kb)

Additional file 2: Description of the physico-chemical quality of cold water (**Table S1.**) and hot water (**Table S2.**) in DWDSs A–E. (PDF 324 kb)

Additional file 3: Number of reads in the analyzed 18S rRNA gene (rDNA) and rRNA transcript (rRNA) sequence libraries (**Table S3.**). (PDF 265 kb)

Additional file 4: Average relative abundances of the most abundant total (DNA) eukaryotic taxa (**Figure S1.**) See active (RNA) eukaryotic taxa (Fig. 2) for legend. (PDF 264 kb)

Additional file 5: Top 50 operational taxonomic units (OTUs) in DWDS A (**Table S4.**), DWDS B (**Table S5.**), DWDS C (**Table S6.**), DWDS D (**Table S7.**) and DWDS E (**Table S8.**) (PDF 223 kb)

Additional file 6: Boxplot of alpha-diversity Shannon values grouped by cold water, hot water, and biofilms in each DWDSs A–E (DNA + RNA) (**Figure S2**). Boxplot of alpha-diversity Shannon values in cold water (**Figure S3**). (PDF 255 kb)

Additional file 7: Heatmap clustering analysis by activity recovery (%) of 66 *Amoebozoa* OTUs (**Figure S4.**), 139 *Fungi* OTUs (**Figure S5.**), 110 *Metazoa* (animal) OTUs (**Figure S6.**), 84 *Chloroplastida* OTUs (Figure S7), 234 *Alveolata* OTUs (**Figure S8.**), 148 *Stramenopiles* OTUs (**Figure S9.**), and 127 *Rhizaria* OTUs (**Figure S10.**). Red color represents a high activity recovery, i.e., relative abundance (%) is higher in RNA than DNA; blue color represents a low activity recovery. (PDF 613 kb)

Additional file 8: Possible pathogenic or opportunistic eukaryotes and free-living amoeba in cold water (**Table S9.**), hot water (**Table S10.**), and biofilms (**Table S11.**) (PDF 271 kb)

Additional file 9: Eukaryotic community (DNA) canonical correspondence analysis (CCA) (**Figure S11.**) (PDF 454 kb)

Additional file 10: Metadata file of samples (**Table S12.**) and DNA/RNA pairs (**Table S13.**) (XLSX 50 kb)

## Data Availability

The datasets generated during the current study including sequences are available in the Short Read Archive (SRA) of NCBI under BioSample accession numbers from SAMN10653499 to SAMN10653948 of the BioProject PRJNA509718. Samples reported in this study account accession numbers SAMN10653499 to SAMN10653784. All samples and controls are listed in a metadata file (Additional file 10: Table S12). The pairs list for corresponding DNA and RNA for each sample is available (Additional file 10: Table S13). An analysis script for the sequence processing, activity recovery calculations, and CCA analysis is available in GitHub under https://github.com/thl-kuopio/DWDSOME-Eukaryote-analysis.

## Abbreviations

AOC: Assimilable organic carbon
DAPI: Total cell count determined using 4,6-diamidino-2-phenylindole staining
DEUF: Dead-end ultrafiltration
DWDS: Drinking water distribution system
EC: Electric conductivity
FLA: Free-living amoebae
HPC: Heterotrophic plate count
MAP: Microbially available phosphorus
NA: Nucleic acid
NMDS: Non-metric multidimensional scaling plot
OTU: Operational taxonomic unit
rDNA: Ribosomal RNA gene (DNA)
rRNA: Ribosomal RNA (RNA)
WM: Water meter
