# Supplementary figures and images for: Active eukaryotes in drinking water distribution systems of ground and surface waterworks
Source: Microbiome. 2019 Jul 3;7:99. doi: 10.1186/s40168-019-0715-5 (PMC6610866; doi:10.1186/s40168-019-0715-5)

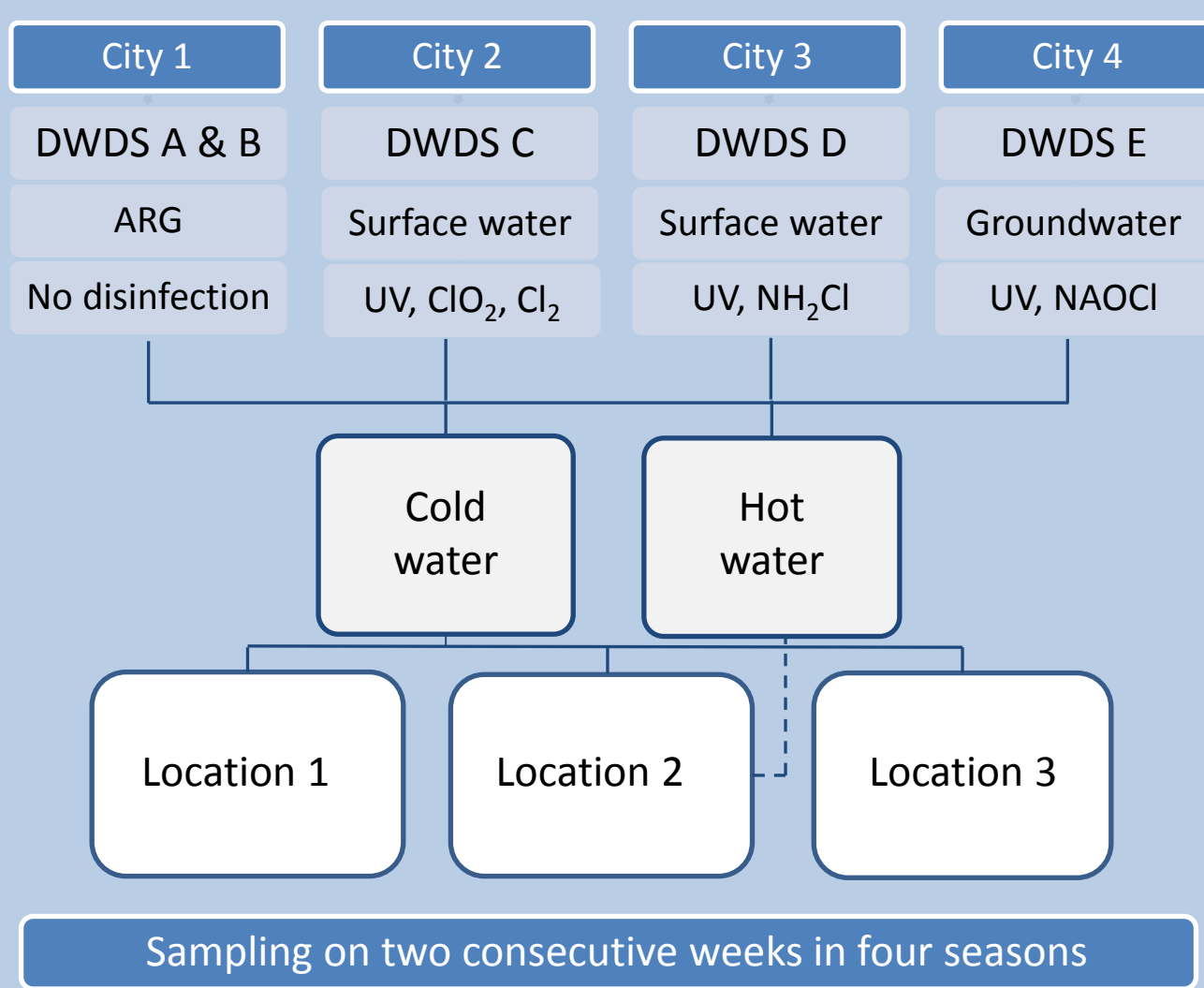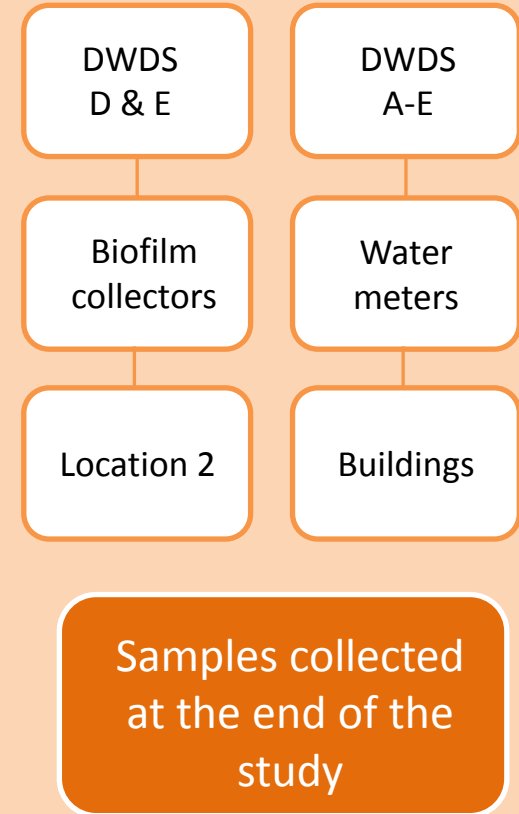

Supplement: Supplementary file 1 — A chart of the experimental design of the study. (PDF 100 kb) [file 40168_2019_715_MOESM1_ESM.pdf]

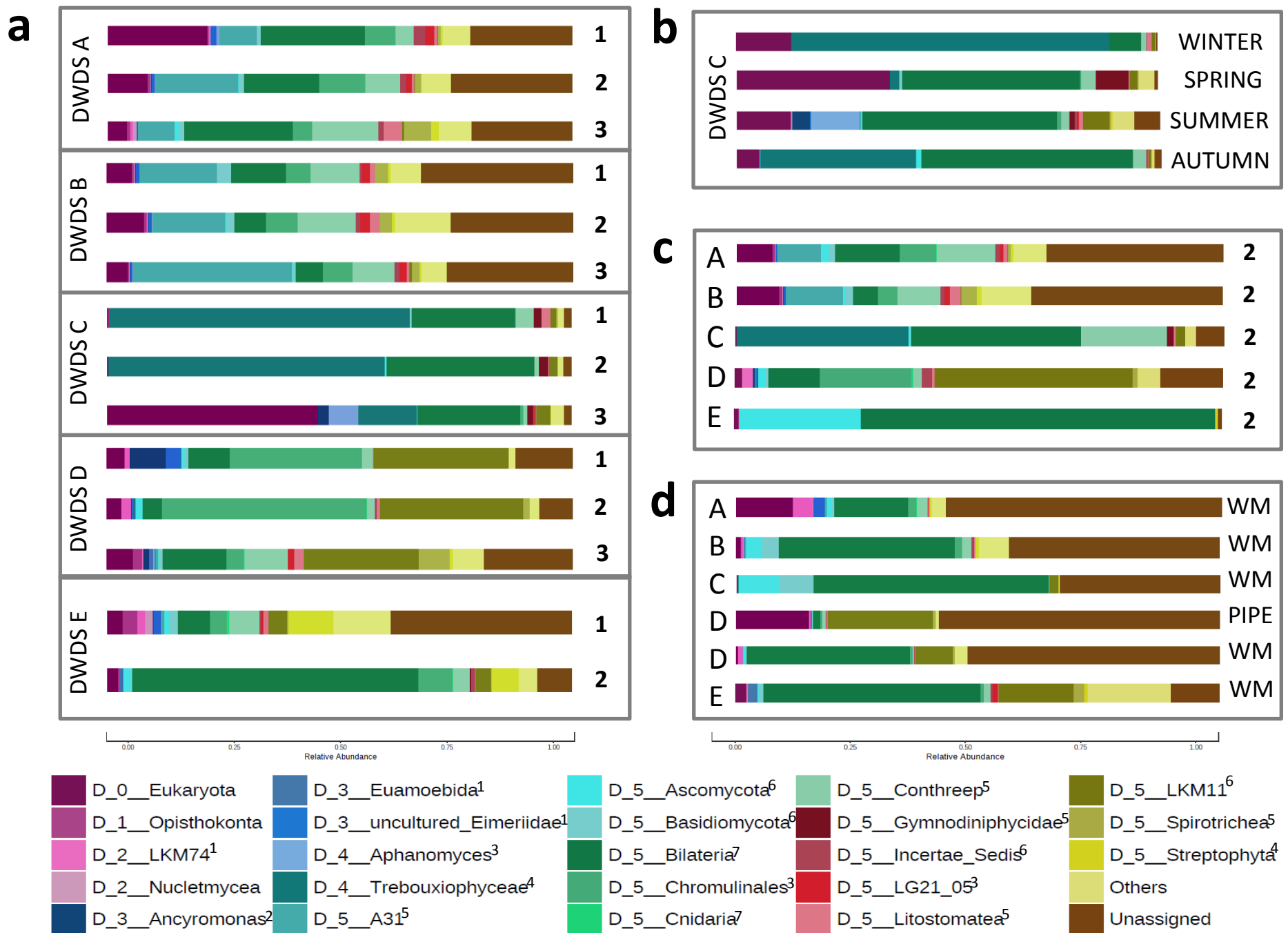

Supplement: Supplementary file 4 — Average relative abundances of the most abundant total (DNA) eukaryotic taxa (Figure S1.) See active (RNA) eukaryotic taxa (Fig. 2) for legend. (PDF 264 kb) [file 40168_2019_715_MOESM4_ESM.pdf]
