# Supplementary material for: Active eukaryotes in drinking water distribution systems of ground and surface waterworks
Source: Microbiome. 2019 Jul 3;7:99. doi: 10.1186/s40168-019-0715-5 (PMC6610866; doi:10.1186/s40168-019-0715-5)
Supplement: Supplementary file 2 — Description of the physico-chemical quality of cold water (Table S1.) and hot water (Table S2.) in DWDSs A–E. (PDF 324 kb) [file 40168_2019_715_MOESM2_ESM.pdf]

Description of physico-chemical and microbiological quality of water

Methods for physico-chemical and microbiological parameters for cold water are described in Ikonen et al. (2017).

Statistically significant differences ($P < 0.05$) between DWDSs occurred nearly in all water quality parameters in cold water (Ikonen et al., 2017). This was notable with respect to some microbial nutrients, *i.e.* total assimilable organic carbon (AOC) was higher in DWDS C and D distributing water from surface waterworks (average 138–143 µg-C/l) than DWDS A, B and E distributing ground water (average 73–89 µg-C/l) and higher microbially available phosphorus (MAP) concentrations occurred in DWDS E (average 4.8 µg-PO_4-P/l) than other DWDS A–D (average ≤ 0.8 µg-PO_4-P/l) (Table S1).

Total chlorine in disinfected DWDS was highest in DWDS C (average 0.3 mg/l) followed by DWDS E (0.2 mg/l) and DWDS D (0.1 mg/l) (Table S1) (Ikonen et al., 2017). Average turbidity, absorbance 254 nm and EC were higher in DWDS A and B than in DWDS C-E (Table S1).

Total cell count (DAPI) in cold water was higher in unchlorinated DWDS A and B (average 1.6–1.9 $\cdot 10^5$ cells/ml) than in chlorinated DWDS C–E (average 3.4–8.3 $\cdot 10^4$ cells/ml).

Reference:

Ikonen JM, Hokajärvi A-M, Heikkinen J, Pitkänen T, Kolehmainen M, Pursiainen A, et al. Drinking water quality in distribution systems of surface and ground waterworks in Finland. J Water Secur. 2017;3:1–10.

Table S1. Cold water quality parameters in DWDS A–E.

Cold water quality parameters	A				B				C				D				E			
	Min	Max	Average	STD	Min	Max	Average	STD	Min	Max	Average	STD	Min	Max	Average	STD	Min	Max	Average	STD
Turbidity NTU	0.01	0.36	0.14	0.09	0.04	0.72	0.11	0.13	0.01	0.11	0.04	0.03	0.02	0.34	0.08	0.06	0.01	0.05	0.02	0.02
Absorbance 254nm	0.17	0.26	0.21	0.02	0.20	0.26	0.23	0.02	0.01	0.18	0.11	0.04	0.07	0.12	0.09	0.01	0.03	0.04	0.04	0.00
Absorbance 420nm	0.01	0.16	0.02	0.03	0.01	0.10	0.02	0.03	0.00	0.02	0.00	0.00	0.00	0.01	0.01	0.00	0.00	0.00	0.00	0.00
Temperature (°C)	5.5	18.1	10.8	3.7	5.8	12.7	9.6	2.3	1.7	20.1	10.3	5.2	5.0	16.6	9.7	3.4	4.0	10.6	6.4	2.0
Cu (mg/l)	0.02	0.08	0.04	0.02	0.02	0.08	0.04	0.02	0.02	0.11	0.05	0.03	0.02	0.08	0.04	0.02	0.02	0.06	0.03	0.01
Al (mg/l)	0.00	0.03	0.01	0.01	0.00	0.02	0.01	0.00	0.00	0.04	0.01	0.01	0.00	0.05	0.01	0.01	0.00	0.03	0.01	0.01
Mn (mg/l)	0.00	0.12	0.02	0.02	0.00	0.01	0.01	0.00	0.00	0.05	0.01	0.01	0.00	0.04	0.01	0.01	0.00	0.01	0.00	0.00
Fe (mg/l)	0.03	0.16	0.07	0.03	0.01	0.09	0.05	0.02	0.01	0.12	0.03	0.02	0.05	0.14	0.09	0.02	0.01	0.17	0.03	0.04
EC (µS/cm)	163	252	217	18	160	240	214	18	145	155	151	3	134	194	147	11	94	160	102	13
pH	7.1	8.6	8.3	0.3	7.2	8.8	8.3	0.3	7.8	9.0	8.3	0.3	7.1	8.7	7.9	0.4	6.2	8.9	8.0	0.7
Total Cl (mg/l)	-	-	-	-	-	-	-	-	0.07	0.61	0.31	0.16	0.02	0.43	0.09	0.11	0.06	0.48	0.19	0.10
Free Cl (mg/l)	-	-	-	-	-	-	-	-	0.03	0.97	0.29	0.24	0.02	0.23	0.08	0.08	0.01	0.33	0.16	0.09
AOC (µg-C/l)	37	175	89	39	39	192	82	40	74	254	143	44	58	257	138	52	9	266	73	63
MAP (µg-PO_4-P/l)	0.4	1.3	0.8	0.3	0.4	1.6	0.8	0.3	0.04	0.9	0.2	0.3	0.1	0.5	0.2	0.1	3.3	6.9	4.8	0.9

AOC: assimilable organic carbon, MAP: microbially available phosphorus, EC: electric conductivity.

Sample and method description: Ikonen JM, Hokajärvi A-M, Heikkinen J, Pitkänen T, Kolehmainen M, Pursiainen A, et al. Drinking water quality in distribution systems of surface and ground waterworks in Finland. J Water Secur. 2017;3:1–10.

Table S2. Hot water quality parameters in DWDS A–E.

Hot water quality parameters	A*				B**				C				D				E			
	Min	Max	Average	STD	Min	Max	Average	STD	Min	Max	Average	STD	Min	Max	Average	STD	Min	Max	Average	STD
Turbidity NTU	0.06	2.83	0.52	0.88	0.01	0.19	0.08	0.05	0.01	0.08	0.03	0.02	0.02	0.34	0.14	0.09	0.01	0.05	0.02	0.01
Absorbance 254nm	0.22	0.49	0.27	0.09	0.21	0.25	0.22	0.01	0.01	0.16	0.12	0.05	0.11	0.16	0.14	0.02	0.04	0.05	0.04	0.00
Absorbance 420nm	0.01	0.02	0.01	0.00	0.01	0.11	0.02	0.03	0.00	0.00	0.00	0.00	0.01	0.02	0.01	0.00	0.00	0.00	0.00	0.00
Temperature (°C)	50.8	56.4	53.8	2.1	52.7	61.9	57.7	3.1	42.9	53.7	50.6	3.6	50.7	58.0	54.6	2.6	48.7	59.3	54.1	3.8
Cu (mg/l)	0.02	0.17	0.08	0.04	0.06	0.08	0.07	0.01	0.07	0.16	0.12	0.03	0.17	0.31	0.24	0.05	0.02	0.02	0.02	0.00
Al (mg/l)	0.00	0.03	0.01	0.01	0.00	0.02	0.01	0.01	0.00	0.03	0.01	0.01	0.00	0.05	0.02	0.01	0.00	0.03	0.02	0.01
Mn (mg/l)	0.00	0.10	0.03	0.03	0.00	0.01	0.01	0.00	0.00	0.03	0.01	0.01	0.00	0.03	0.01	0.01	0.00	0.01	0.00	0.00
Fe (mg/l)	0.04	1.11	0.19	0.35	0.03	0.07	0.05	0.02	0.01	0.04	0.02	0.01	0.06	0.13	0.10	0.02	0.01	0.02	0.01	0.00
EC (µS/cm)	215	364	244	46	185	248	220	17	149	156	152	2	140	151	145	4	98	111	104	4
pH	8.0	8.3	8.1	0.1	7.7	8.5	8.1	0.2	7.8	8.5	8.0	0.2	7.4	8.0	7.7	0.2	8.0	8.7	8.3	0.2

**In one sample (DWDS A, autumn, first sampling week) exceptionally high values of Fe (1.1 mg/l), Mn (0.1 mg/l), turbidity (2.8 NTU), absorbance 254nm (0.5) and SPC (360µS/cm).*
***Exceptionally high Absorbance 420 nm (0.1) (DWDS B, spring, second sampling week).*
AOC (assimilable organic carbon) and MAP (microbially available phosphorus) = not analyzed.
EC: electric conductivity.
Total chlorine and free chlorine analyzed only sporadically: 0 to 0.2 mg/l.
Method description: Ikonen JM, Hokajärvi A-M, Heikkinen J, Pitkänen T, Kolehmainen M, Pursiainen A, et al. Drinking water quality in distribution systems of surface and ground waterworks in Finland. J Water Secur. 2017;3:1–10.
