# Supplementary material for: Active eukaryotes in drinking water distribution systems of ground and surface waterworks
Source: Microbiome. 2019 Jul 3;7:99. doi: 10.1186/s40168-019-0715-5 (PMC6610866; doi:10.1186/s40168-019-0715-5)
Supplement: Supplementary file 3 — Number of reads in the analyzed 18S rRNA gene (rDNA) and rRNA transcript (rRNA) sequence libraries (Table S3.). (PDF 265 kb) [file 40168_2019_715_MOESM3_ESM.pdf]

Table S3. Number of reads in the analysed 18S rRNA gene (rDNA) and rRNA transcript (rRNA) sequence libraries.

					DNA					RNA				
Sample type	DWDS*	Location: Distance from	Season**	Sampling event details	N (analysed)	N (excluded)	Average reads	Min reads	Max reads	N (analysed)	N (excluded)	Average reads	Min reads	Max reads
Cold water 100L	A	Three DWDS locations 1: 1-9 km 2: 3-26 km 3: 11-36 km	Four seasons (winter, spring, summer, autumn)	Two consecutive sampling weeks (1 and 2)	23	1	19 000	3 500	79 000	24	0	31 000	7 000	170 000
	B				23	1	18 000	6 900	45 000	24	0	26 000	9 000	56 000
	C				22	0	25 000	3 100	86 000	21	1	45 000	8 800	99 000
	D				24	0	25 000	6 300	53 000	23	1	30 000	6 500	76 000
	E				6	19	17 000	3 200	39 000	20	5	22 000	3 800	50 000
Hot water 100L	A	One DWDS location 2: 3-26 km	Four seasons (winter, spring, summer, autumn)	Two consecutive sampling weeks (1 and 2)	5	3	19 000	3 600	54 000	5	3	10 000	2 900	17 000
	B				8	0	12 000	3 000	26 000	6	2	12 000	4 400	34 000
	C				5	3	9 300	5 200	14 000	2	6	5 100	4 000	6 100
	D				7	1	9 200	3 300	29 000	3	5	5 100	4 300	5 700
	E				1	7	NA	6 600	NA	1	7	NA	6 000	NA
Pipeline biofilm	D	One location 2: 3-26 km	Late autumn	Age of biofilm approx. 9 months	3	0	21 000	8 200	39 000	3	0	24 000	16 000	30 000
	E				0	3	NA	NA	NA	0	3	NA	NA	NA
Watermeter soft deposit	A	Two buildings: NA	Late autumn	5.4 - 13.8 years	2	0	16 000	12 000	21 000	2	0	14 000	13 000	15 000
	B	Two buildings: NA		6.8 - 9.9. years	2	0	7 900	3 800	12 000	2	0	13 000	12 000	14 000
	C	Two buildings: NA		4.6 - 9 years	1	1	NA	7 900	NA	2	0	6 300	2 900	9 600
	D	One building: NA		7.8 years	1	0	NA	38 000	NA	1	0	NA	36 000	NA
	E	Two buildings: NA		15 - 19 years	2	1	29 000	28 000	30 000	3	0	29 000	11 000	44 000
Water-meter water	A	Two buildings: NA	Late autumn	One sampling week	2	0	15 000	9 300	20 000	1	1	NA	45 000	NA
	B	Two buildings: NA			2	0	22 000	14 000	29 000	2	0	17 000	15 000	19 000
	C	One building: NA			1	0	NA	12 000	NA	1	0	NA	12 000	NA
Sum					140	40	2 600 000			146	34	4 000 000		

**(A-B) = No disinfection, artificial groundwater; C = UV-light + chlorine dioxide, surface water; D = UV-light + monochloramine, surface water; E = UV-light + sodium hypochlorite, groundwater.*

***Winter = January-March 2015, spring = April-May 2015, summer = August-September 2015, autumn = October-December 2015.*

N = number of samples. Excluded samples contained reads below rarefraction 2873. Average, min and max values calculated from analysed samples. NA = Not applicable.
