# Supplementary material for: Active eukaryotes in drinking water distribution systems of ground and surface waterworks
Source: Microbiome. 2019 Jul 3;7:99. doi: 10.1186/s40168-019-0715-5 (PMC6610866; doi:10.1186/s40168-019-0715-5)
Supplement: Supplementary file 5 — Top 50 operational taxonomic units (OTUs) in DWDS A (Table S4.), DWDS B (Table S5.), DWDS C (Table S6.), DWDS D (Table S7.) and DWDS E (Table S8.) (PDF 223 kb) [file 40168_2019_715_MOESM5_ESM.pdf]

Table S4. TOP 50 Operational taxonomic units (OTUs) in DWDS A.

OTU ID	Taxonomic classification	% ALL	%DNA	%RNA	%cold water	%hot water	%biofilms
KJ925353.1.1495	D_0__Eukaryota	3.5	5.1	1.9	3.8	3.5	0.1
KC188996.1.1829	D_0__Eukaryota	0.8	0.7	0.9	0.2	0.3	9.9
EF024971.1.2014	D_0__Eukaryota; D_1__Amoebozoa; D_2__LEMD255; D_3__uncultured Eimeriidae	1.4	0.8	1.9	1.4	0.2	3.1
AY919786.1.1863	D_0__Eukaryota; D_1__Amoebozoa; D_2__LKM74	1.0	0.4	1.5	0.7	0.0	7.1
New.ReferenceOTU782	D_0__Eukaryota; D_1__Archaeplastida; D_2__Chloroplastida; D_3__Charophyta; D_4__Phragmoplastophyta; D_5__Streptophyta; D_6__Embryophyta; D_7__Tracheophyta; D_8__Spermatophyta; D_9__Magnoliophyta; D_10__Brassicales; D_11__Cleome; D_12__Tarenaya hassleriana	1.0	0.2	1.9	1.3	0.00	0.00
New.ReferenceOTU1021	D_0__Eukaryota; D_1__Archaeplastida; D_2__Chloroplastida; D_3__Charophyta; D_4__Phragmoplastophyta; D_5__Streptophyta; D_6__Embryophyta; D_7__Tracheophyta; D_8__Spermatophyta; D_9__Magnoliophyta; D_10__Brassicales; D_11__Cleome; D_12__Tarenaya hassleriana	0.5	0.0	1.1	0.5	0.0	2.1
New.ReferenceOTU680	D_0__Eukaryota; D_1__Opisthokonta	1.5	0.2	2.8	1.7	0.8	0.8
FJ577820.1.1699	D_0__Eukaryota; D_1__Opisthokonta; D_2__Holozoa; D_3__Metazoa (Animalia); D_4__Eumetazoa; D_5__Bilateria; D_6__Annelida; D_7__Clitellata; D_8__Oligochaeta; D_9__Haplotaxida	1.3	1.3	1.3	1.1	3.1	0.1
New.ReferenceOTU1146	D_0__Eukaryota; D_1__Opisthokonta; D_2__Holozoa; D_3__Metazoa (Animalia); D_4__Eumetazoa; D_5__Bilateria; D_6__Arthropoda; D_7__Crustacea; D_8__Maxillopoda; D_9__Copepoda	1.2	2.0	0.4	1.4	0.8	0.00
AY627015.1.1809	D_0__Eukaryota; D_1__Opisthokonta; D_2__Holozoa; D_3__Metazoa (Animalia); D_4__Eumetazoa; D_5__Bilateria; D_6__Arthropoda; D_7__Crustacea; D_8__Maxillopoda; D_9__Copepoda	0.5	1.0	0.01	0.03	3.1	0.00
ABRN02315288.22.1468	D_0__Eukaryota; D_1__Opisthokonta; D_2__Holozoa; D_3__Metazoa (Animalia); D_4__Eumetazoa; D_5__Bilateria; D_6__Chordata; D_7__Vertebrata; D_8__Gnathostomata; D_9__Euteleostomi; D_10__Tetrapoda; D_11__Mammalia	0.6	0.8	0.5	0.4	2.2	0.1
AJ130867.1.1626	D_0__Eukaryota; D_1__Opisthokonta; D_2__Holozoa; D_3__Metazoa (Animalia); D_4__Eumetazoa; D_5__Bilateria; D_6__Gastrotricha; D_7__Chaetonotida	1.5	3.0	0.1	1.9	0.1	0.00
KF159023.1.1819	D_0__Eukaryota; D_1__Opisthokonta; D_2__Holozoa; D_3__Metazoa (Animalia); D_4__Eumetazoa; D_5__Bilateria; D_6__Gastrotricha; D_7__Chaetonotida	0.9	1.6	0.1	1.1	0.1	0.00
New.ReferenceOTU365	D_0__Eukaryota; D_1__Opisthokonta; D_2__Holozoa; D_3__Metazoa (Animalia); D_4__Eumetazoa; D_5__Bilateria; D_6__Hemichordata; D_7__Enteropneusta; D_8__Harrimaniidae	0.5	0.5	0.4	0.6	0.03	0.00
EF024023.1.1780	D_0__Eukaryota; D_1__Opisthokonta; D_2__Holozoa; D_3__Metazoa (Animalia); D_4__Eumetazoa; D_5__Bilateria; D_6__Nematoda; D_7__Chromadorea; D_8__Monhysterida	0.7	1.0	0.5	0.9	0.2	0.1
AY284729.1.1645	D_0__Eukaryota; D_1__Opisthokonta; D_2__Holozoa; D_3__Metazoa (Animalia); D_4__Eumetazoa; D_5__Bilateria; D_6__Nematoda; D_7__Enoplea; D_8__Enoplia; D_9__Triplonchida	0.7	1.2	0.2	0.2	0.8	7.5
New.ReferenceOTU367	D_0__Eukaryota; D_1__Opisthokonta; D_2__Holozoa; D_3__Metazoa (Animalia); D_4__Eumetazoa; D_5__Bilateria; D_6__Platyhelminthes; D_7__Monogenea; D_8__Monopisthocotylea; D_9__Gyrodactylidae	0.5	0.3	0.8	0.7	0.1	0.03
GACQ01020207.292.2090	D_0__Eukaryota; D_1__Opisthokonta; D_2__Holozoa; D_3__Metazoa (Animalia); D_4__Eumetazoa; D_5__Bilateria; D_6__Rotifera; D_7__Monogononta	1.2	0.6	1.7	1.5	0.03	0.00
GU070888.1.1815	D_0__Eukaryota; D_1__Opisthokonta; D_2__Holozoa; D_3__Metazoa (Animalia); D_4__Eumetazoa; D_5__Bilateria; D_6__Rotifera; D_7__Monogononta; D_8__Flosculariacea	0.6	0.6	0.6	0.8	0.05	0.01
New.ReferenceOTU110	D_0__Eukaryota; D_1__Opisthokonta; D_2__Nucletmycea	1.4	0.3	2.5	1.6	0.5	1.1
JN705505.1.1364	D_0__Eukaryota; D_1__SAR; D_2__Alveolata; D_3__Ciliophora; D_4__Intramacronucleata; D_5__Conthreep; D_6__Oligohymenophorea; D_7__CV1-2A-17	0.5	0.4	0.6	0.6	0.1	0.3
New.ReferenceOTU687	D_0__Eukaryota; D_1__SAR; D_2__Alveolata; D_3__Ciliophora; D_4__Intramacronucleata; D_5__Conthreep; D_6__Oligohymenophorea; D_7__Hymenostomatia	2.2	2.7	1.7	2.3	2.9	0.1
New.ReferenceOTU265	D_0__Eukaryota; D_1__SAR; D_2__Alveolata; D_3__Ciliophora; D_4__Intramacronucleata; D_5__Conthreep; D_6__Phyllopharyngea; D_7__Cyrtophoria; D_8__Chilodonella	1.4	0.9	1.8	1.6	0.6	0.0
New.ReferenceOTU183	D_0__Eukaryota; D_1__SAR; D_2__Alveolata; D_3__Ciliophora; D_4__Intramacronucleata; D_5__Conthreep; D_6__Phyllopharyngea; D_7__Cyrtophoria; D_8__Trochilioides; D_9__uncultured eukaryote	1.6	1.2	2.1	2.0	0.3	0.00
AB505474.1.1697	D_0__Eukaryota; D_1__SAR; D_2__Alveolata; D_3__Ciliophora; D_4__Intramacronucleata; D_5__Spirotrichea; D_6__Hypotrichia; D_7__Holosticha	1.0	1.8	0.2	1.2	0.3	0.01
New.ReferenceOTU1154	D_0__Eukaryota; D_1__SAR; D_2__Alveolata; D_3__Protalveolata; D_4__Perkinsidae; D_5__A31; D_6__uncultured alveolate	1.4	2.3	0.5	1.6	1.0	0.1
New.ReferenceOTU374	D_0__Eukaryota; D_1__SAR; D_2__Alveolata; D_3__Protalveolata; D_4__Perkinsidae; D_5__A31; D_6__uncultured alveolate	0.5	0.8	0.3	0.7	0.1	0.1
New.ReferenceOTU896	D_0__Eukaryota; D_1__SAR; D_2__Alveolata; D_3__Protalveolata; D_4__Perkinsidae; D_5__A31; D_6__uncultured freshwater eukaryote	3.4	6.8	0.1	3.8	2.9	0.02
DQ388541.1.1646	D_0__Eukaryota; D_1__SAR; D_2__Stramenopiles; D_3__Ochrophyta; D_4__Chrysophyceae; D_5__Chromulinales; D_6__Spumella	3.3	4.7	1.9	3.0	5.5	1.1
New.ReferenceOTU688	D_0__Eukaryota; D_1__SAR; D_2__Stramenopiles; D_3__Ochrophyta; D_4__Chrysophyceae; D_5__Chromulinales; D_6__Spumella	0.7	1.0	0.4	0.6	1.5	0.02
AB520724.1.1665	D_0__Eukaryota; D_1__SAR; D_2__Stramenopiles; D_3__Ochrophyta; D_4__Chrysophyceae; D_5__Chromulinales; D_6__Spumella	0.5	0.7	0.4	0.5	0.9	0.02
New.ReferenceOTU1122	D_0__Eukaryota; D_1__SAR; D_2__Stramenopiles; D_3__Ochrophyta; D_4__Chrysophyceae; D_5__LG21-05; D_6__uncultured eukaryote	0.9	0.4	1.4	1.1	0.3	0.03
New.ReferenceOTU1108	D_0__Eukaryota; D_1__SAR; D_2__Stramenopiles; D_3__Ochrophyta; D_4__Chrysophyceae; D_5__P34.45	0.6	0.3	0.8	0.5	1.0	0.1
New.ReferenceOTU1158	Unassigned	3.4	6.8	0.0	2.9	7.2	0.4
New.ReferenceOTU150	Unassigned	2.2	0.2	4.2	2.4	2.1	0.01
New.ReferenceOTU497	Unassigned	2.2	3.4	1.0	0.2	0.6	30.8
New.ReferenceOTU378	Unassigned	1.4	2.4	0.3	1.2	1.3	4.1
New.ReferenceOTU98	Unassigned	1.3	0.3	2.3	1.7	0.03	0.00
New.ReferenceOTU73	Unassigned	1.2	0.9	1.6	1.4	0.4	0.9
New.ReferenceOTU278	Unassigned	0.9	1.5	0.2	0.9	1.1	0.2
New.ReferenceOTU992	Unassigned	0.8	1.5	0.2	0.8	1.4	0.2
New.ReferenceOTU282	Unassigned	0.8	0.2	1.4	0.5	2.6	0.03
New.ReferenceOTU742	Unassigned	0.7	0.3	1.2	0.9	0.3	0.1
New.ReferenceOTU957	Unassigned	0.7	0.2	1.2	0.8	0.2	0.00
New.ReferenceOTU847	Unassigned	0.7	0.2	1.1	0.8	0.3	0.02
New.ReferenceOTU854	Unassigned	0.6	0.0	1.3	0.8	0.1	0.2
New.ReferenceOTU654	Unassigned	0.6	0.2	1.0	0.6	0.3	0.9
New.ReferenceOTU1024	Unassigned	0.5	0.6	0.4	0.4	1.1	0.1
New.ReferenceOTU777	Unassigned	0.5	0.1	0.9	0.6	0.1	0.0
New.ReferenceOTU71	Unassigned	0.5	0.4	0.6	0.4	1.2	0.2

Samples (%ALL, average abundance from 64 samples) grouped by template (DNA/RNA) and water system properties (cold water/hot water/biofilm).

Table S5. TOP 50 Operational taxonomic units (OTUs) in DWDS B.

OTU ID	Taxonomic classification	% ALL	%DNA	%RNA	%cold water	%hot water	%biofilms
KJ925353.1.1495	D_0__Eukaryota	2.2	3.0	1.3	2.0	3.4	0.1
AB901824.1.1748	D_0__Eukaryota	0.6	1.0	0.1	0.6	0.5	0.0
KC188996.1.1829	D_0__Eukaryota	0.5	0.8	0.1	0.1	1.9	0.0
EF024971.1.2014	D_0__Eukaryota; D_1__Amoebozoa; D_2__LEMD255; D_3__uncultured Eimeriidae	1.1	0.6	1.7	1.4	0.6	0.2
New.ReferenceOTU1021	D_0__Eukaryota; D_1__Archaeplastida; D_2__Chloroplastida; D_3__Charophyta; D_4__Phragmoplastophyta; D_5__Streptophyta; D_6__Embryophyta; D_7__Tracheophyta; D_8__Spermatophyta; D_9__Magnoliophyta; D_10__Brassicales; D_11__Cleome; D_12__Tarenaya hassleriana	10.8	0.0	21.9	12.8	3.4	10.5
New.ReferenceOTU680	D_0__Eukaryota; D_1__Opisthokonta	0.7	0.1	1.2	0.7	0.2	1.4
FJ577820.1.1699	D_0__Eukaryota; D_1__Opisthokonta; D_2__Holozoa; D_3__Metazoa (Animalia); D_4__Eumetazoa; D_5__Bilateria; D_6__Annelida; D_7__Clitellata; D_8__Oligochaeta; D_9__Haplotaxida	1.1	1.5	0.8	0.2	0.1	17.2
New.ReferenceOTU1146	D_0__Eukaryota; D_1__Opisthokonta; D_2__Holozoa; D_3__Metazoa (Animalia); D_4__Eumetazoa; D_5__Bilateria; D_6__Arthropoda; D_7__Crustacea; D_8__Maxillopoda; D_9__Copepoda	0.8	1.4	0.1	1.0	0.0	0.0
ABRN02315288.22.1468	D_0__Eukaryota; D_1__Opisthokonta; D_2__Holozoa; D_3__Metazoa (Animalia); D_4__Eumetazoa; D_5__Bilateria; D_6__Chordata; D_7__Vertebrata; D_8__Gnathostomata; D_9__Euteleostomi; D_10__Tetrapoda; D_11__Mammalia	0.4	0.4	0.3	0.1	1.4	0.6
AJ130867.1.1626	D_0__Eukaryota; D_1__Opisthokonta; D_2__Holozoa; D_3__Metazoa (Animalia); D_4__Eumetazoa; D_5__Bilateria; D_6__Gastrotricha; D_7__Chaetonotida	1.0	1.8	0.1	1.3	0.0	0.0
EF024023.1.1780	D_0__Eukaryota; D_1__Opisthokonta; D_2__Holozoa; D_3__Metazoa (Animalia); D_4__Eumetazoa; D_5__Bilateria; D_6__Nematoda; D_7__Chromadorea; D_8__Monhysterida	0.6	0.9	0.2	0.6	0.7	0.1
New.ReferenceOTU110	D_0__Eukaryota; D_1__Opisthokonta; D_2__Nucletmycea	0.7	0.1	1.3	0.8	0.3	0.4
New.ReferenceOTU16	D_0__Eukaryota; D_1__Opisthokonta; D_2__Nucletmycea; D_3__Fungi; D_4__Dikarya; D_5__Basidiomycota; D_6__Agaricomycotina; D_7__Agaricomycetes; D_8__Polyporales; D_9__Polyporaceae; D_10__Coriolopsis; D_11__Coriolopsis gallica	0.4	0.4	0.3	0.4	0.3	0.0
JN705505.1.1364	D_0__Eukaryota; D_1__SAR; D_2__Alveolata; D_3__Ciliophora; D_4__Intramacronucleata; D_5__Conthreep; D_6__Oligohymenophorea; D_7__CV1-2A-17	0.5	0.3	0.6	0.5	0.1	1.2
New.ReferenceOTU687	D_0__Eukaryota; D_1__SAR; D_2__Alveolata; D_3__Ciliophora; D_4__Intramacronucleata; D_5__Conthreep; D_6__Oligohymenophorea; D_7__Hymenostomatia	2.4	2.8	2.0	2.4	3.1	0.2
AY821924.1.1542	D_0__Eukaryota; D_1__SAR; D_2__Alveolata; D_3__Ciliophora; D_4__Intramacronucleata; D_5__Conthreep; D_6__Oligohymenophorea; D_7__Hymenostomatia	0.4	0.4	0.3	0.4	0.3	0.0
New.ReferenceOTU325	D_0__Eukaryota; D_1__SAR; D_2__Alveolata; D_3__Ciliophora; D_4__Intramacronucleata; D_5__Conthreep; D_6__Oligohymenophorea; D_7__Peniculia; D_8__Paramecium; D_9__Paramecium duboscqui	0.6	1.1	0.1	0.2	2.0	0.0
New.ReferenceOTU295	D_0__Eukaryota; D_1__SAR; D_2__Alveolata; D_3__Ciliophora; D_4__Intramacronucleata; D_5__Conthreep; D_6__Oligohymenophorea; D_7__Peritrichia	0.8	1.1	0.4	1.0	0.1	0.3
EU446379.1.1533	D_0__Eukaryota; D_1__SAR; D_2__Alveolata; D_3__Ciliophora; D_4__Intramacronucleata; D_5__Conthreep; D_6__Oligohymenophorea; D_7__Scuticociliatia	0.6	0.4	0.7	0.7	0.4	0.0
HM030917.1.1568	D_0__Eukaryota; D_1__SAR; D_2__Alveolata; D_3__Ciliophora; D_4__Intramacronucleata; D_5__Conthreep; D_6__Phyllopharyngea; D_7__Cyrtophoria; D_8__Chilodonella; D_9__Chilodonella uncinata	0.5	0.4	0.6	0.6	0.3	0.0
AJ130860.1.1539	D_0__Eukaryota; D_1__SAR; D_2__Alveolata; D_3__Ciliophora; D_4__Intramacronucleata; D_5__Conthreep; D_6__Phyllopharyngea; D_7__Cyrtophoria; D_8__Trithigmostoma; D_9__uncultured eukaryote	1.1	0.4	1.8	1.4	0.3	0.0
New.ReferenceOTU183	D_0__Eukaryota; D_1__SAR; D_2__Alveolata; D_3__Ciliophora; D_4__Intramacronucleata; D_5__Conthreep; D_6__Phyllopharyngea; D_7__Cyrtophoria; D_8__Trochilioides; D_9__uncultured eukaryote	0.6	0.7	0.5	0.7	0.4	0.0
New.ReferenceOTU1148	D_0__Eukaryota; D_1__SAR; D_2__Alveolata; D_3__Ciliophora; D_4__Intramacronucleata; D_5__Litostomatea; D_6__Haptoria; D_7__Phialina	0.5	0.4	0.5	0.3	1.3	0.0
AB505474.1.1697	D_0__Eukaryota; D_1__SAR; D_2__Alveolata; D_3__Ciliophora; D_4__Intramacronucleata; D_5__Spirotrichea; D_6__Hypotrichia; D_7__Holosticha	0.6	1.1	0.1	0.6	0.7	0.0
New.ReferenceOTU405	D_0__Eukaryota; D_1__SAR; D_2__Alveolata; D_3__Ciliophora; D_4__Postciliodesmatophora; D_5__Heterotrichea	0.6	0.4	0.8	0.3	1.9	0.0
New.ReferenceOTU1154	D_0__Eukaryota; D_1__SAR; D_2__Alveolata; D_3__Protalveolata; D_4__Perkinsidae; D_5__A31; D_6__uncultured alveolate	2.8	5.3	0.2	3.0	3.0	0.0
New.ReferenceOTU896	D_0__Eukaryota; D_1__SAR; D_2__Alveolata; D_3__Protalveolata; D_4__Perkinsidae; D_5__A31; D_6__uncultured freshwater eukaryote	5.6	10.9	0.1	6.8	2.6	0.1
New.ReferenceOTU403	D_0__Eukaryota; D_1__SAR; D_2__Rhizaria; D_3__Cercozoa; D_4__uncultured; D_5__uncultured eukaryote	0.4	0.2	0.6	0.4	0.4	0.0
DQ388541.1.1646	D_0__Eukaryota; D_1__SAR; D_2__Stramenopiles; D_3__Ochrophyta; D_4__Chrysophyceae; D_5__Chromulinales; D_6__Spumella	2.2	3.2	1.2	2.4	2.0	0.6
New.ReferenceOTU688	D_0__Eukaryota; D_1__SAR; D_2__Stramenopiles; D_3__Ochrophyta; D_4__Chrysophyceae; D_5__Chromulinales; D_6__Spumella	0.8	1.1	0.4	0.8	1.1	0.1
AB520724.1.1665	D_0__Eukaryota; D_1__SAR; D_2__Stramenopiles; D_3__Ochrophyta; D_4__Chrysophyceae; D_5__Chromulinales; D_6__Spumella	0.5	0.7	0.3	0.5	0.5	0.0
EF526817.1.1395	D_0__Eukaryota; D_1__SAR; D_2__Stramenopiles; D_3__Ochrophyta; D_4__Chrysophyceae; D_5__LG21-05	0.5	0.5	0.5	0.5	0.9	0.0
New.ReferenceOTU1122	D_0__Eukaryota; D_1__SAR; D_2__Stramenopiles; D_3__Ochrophyta; D_4__Chrysophyceae; D_5__LG21-05; D_6__uncultured eukaryote	0.8	0.3	1.3	0.9	0.7	0.0
New.ReferenceOTU1108	D_0__Eukaryota; D_1__SAR; D_2__Stramenopiles; D_3__Ochrophyta; D_4__Chrysophyceae; D_5__P34.45	0.4	0.2	0.5	0.4	0.3	0.0
New.ReferenceOTU1158	Unassigned	2.8	5.6	0.0	1.0	7.7	8.4
New.ReferenceOTU278	Unassigned	2.1	3.6	0.7	2.4	1.6	0.7
New.ReferenceOTU150	Unassigned	1.7	0.2	3.2	1.7	2.1	0.0
New.ReferenceOTU497	Unassigned	1.4	2.4	0.3	1.5	1.0	0.2
New.ReferenceOTU992	Unassigned	1.3	2.2	0.3	1.1	2.1	0.4
New.ReferenceOTU378	Unassigned	1.2	2.1	0.2	1.0	0.6	4.8
New.ReferenceOTU854	Unassigned	1.1	0.0	2.2	1.5	0.0	0.2
New.ReferenceOTU345	Unassigned	0.8	0.4	1.3	1.1	0.1	0.1
New.ReferenceOTU1024	Unassigned	0.8	0.8	0.7	0.8	0.5	1.0
New.ReferenceOTU282	Unassigned	0.7	0.3	1.1	0.7	0.9	0.1
New.ReferenceOTU654	Unassigned	0.6	0.2	1.0	0.6	0.3	1.9
New.ReferenceOTU742	Unassigned	0.6	0.2	0.9	0.7	0.2	0.4
New.ReferenceOTU824	Unassigned	0.5	0.3	0.8	0.7	0.0	0.6
New.ReferenceOTU73	Unassigned	0.5	0.3	0.7	0.6	0.3	0.3
New.ReferenceOTU847	Unassigned	0.5	0.2	0.8	0.6	0.1	0.0
New.ReferenceOTU514	Unassigned	0.4	0.4	0.4	0.5	0.3	0.1

Samples (%ALL, average abundance from 69 samples) grouped by template (DNA/RNA) and water system properties (cold water/hot water/biofilm).

Table S6. TOP 50 Operational taxonomic units (OTUs) in DWDS C.

OTU ID	Taxonomic classification	% ALL	%DNA	%RNA	%cold water	%hot water	%biofilms
HM486990.1.1343	D_0__Eukaryota	8.4	9.6	7.0	10.2	0.0	0.0
KC188996.1.1829	D_0__Eukaryota	1.0	0.0	2.0	0.0	0.0	17.3
New.ReferenceOTU181	D_0__Eukaryota	0.2	0.0	0.4	0.2	0.0	0.0
AY919786.1.1863	D_0__Eukaryota; D_1__Amoebozoa; D_2__LKM74	0.6	0.0	1.2	0.1	0.0	9.7
X89012.1.2653	D_0__Eukaryota; D_1__Archaeplastida; D_2__Chloroplastida; D_3__Chlorophyta; D_4__Trebouxiophyceae	25.6	18.5	33.5	25.8	33.7	3.0
New.ReferenceOTU612	D_0__Eukaryota; D_1__Archaeplastida; D_2__Chloroplastida; D_3__Chlorophyta; D_4__Trebouxiophyceae	10.8	9.6	12.2	10.5	17.1	1.3
EU349232.1.1720	D_0__Eukaryota; D_1__Incertae Sedis; D_2__Ancyromonadida; D_3__Ancyromonas	0.8	0.6	0.9	0.9	0.0	0.0
JX945137.1.1383	D_0__Eukaryota; D_1__Opisthokonta; D_2__Holozoa; D_3__Metazoa (Animalia); D_4__Eumetazoa; D_5__Bilateria; D_6__Arthropoda; D_7__Crustacea; D_8__Maxillopoda; D_9__Copepoda; D_10__Calanoida	4.4	8.2	0.0	4.1	8.1	0.0
GU969144.1.1761	D_0__Eukaryota; D_1__Opisthokonta; D_2__Holozoa; D_3__Metazoa (Animalia); D_4__Eumetazoa; D_5__Bilateria; D_6__Arthropoda; D_7__Crustacea; D_8__Maxillopoda; D_9__Copepoda; D_10__Calanoida	0.4	0.8	0.0	0.5	0.1	0.0
JX995300.1.1753	D_0__Eukaryota; D_1__Opisthokonta; D_2__Holozoa; D_3__Metazoa (Animalia); D_4__Eumetazoa; D_5__Bilateria; D_6__Arthropoda; D_7__Crustacea; D_8__Maxillopoda; D_9__Copepoda; D_10__Calanoida	0.3	0.5	0.0	0.3	0.1	0.0
JX995303.1.1751	D_0__Eukaryota; D_1__Opisthokonta; D_2__Holozoa; D_3__Metazoa (Animalia); D_4__Eumetazoa; D_5__Bilateria; D_6__Arthropoda; D_7__Crustacea; D_8__Maxillopoda; D_9__Copepoda; D_10__Calanoida	0.2	0.4	0.0	0.3	0.0	0.0
DQ107579.1.1432	D_0__Eukaryota; D_1__Opisthokonta; D_2__Holozoa; D_3__Metazoa (Animalia); D_4__Eumetazoa; D_5__Bilateria; D_6__Arthropoda; D_7__Crustacea; D_8__Maxillopoda; D_9__Copepoda; D_10__Cyclopoida	4.1	6.3	1.7	4.7	2.5	0.0
New.ReferenceOTU395	D_0__Eukaryota; D_1__Opisthokonta; D_2__Holozoa; D_3__Metazoa (Animalia); D_4__Eumetazoa; D_5__Bilateria; D_6__Arthropoda; D_7__Crustacea; D_8__Maxillopoda; D_9__Copepoda; D_10__Cyclopoida	2.9	5.1	0.3	2.5	6.4	0.0
AJ746330.1.1809	D_0__Eukaryota; D_1__Opisthokonta; D_2__Holozoa; D_3__Metazoa (Animalia); D_4__Eumetazoa; D_5__Bilateria; D_6__Arthropoda; D_7__Crustacea; D_8__Maxillopoda; D_9__Copepoda; D_10__Cyclopoida	0.9	1.5	0.2	1.1	0.0	0.0
KC315823.1.1652	D_0__Eukaryota; D_1__Opisthokonta; D_2__Holozoa; D_3__Metazoa (Animalia); D_4__Eumetazoa; D_5__Bilateria; D_6__Arthropoda; D_7__Crustacea; D_8__Maxillopoda; D_9__Copepoda; D_10__Cyclopoida	0.4	0.6	0.1	0.4	0.1	0.0
New.ReferenceOTU312	D_0__Eukaryota; D_1__Opisthokonta; D_2__Holozoa; D_3__Metazoa (Animalia); D_4__Eumetazoa; D_5__Bilateria; D_6__Arthropoda; D_7__Crustacea; D_8__Maxillopoda; D_9__Copepoda; D_10__Cyclopoida	0.3	0.7	0.0	0.2	1.3	0.0
ABRN02315288.22.1468	D_0__Eukaryota; D_1__Opisthokonta; D_2__Holozoa; D_3__Metazoa (Animalia); D_4__Eumetazoa; D_5__Bilateria; D_6__Chordata; D_7__Vertebrata; D_8__Gnathostomata; D_9__Euteleostomi; D_10__Tetrapoda; D_11__Mammalia	0.8	1.2	0.3	0.6	1.2	3.1
AJ875123.1.1729	D_0__Eukaryota; D_1__Opisthokonta; D_2__Holozoa; D_3__Metazoa (Animalia); D_4__Eumetazoa; D_5__Bilateria; D_6__Nematoda; D_7__Chromadorea; D_8__Araeolaimida	1.1	1.8	0.4	0.0	0.7	18.4
New.ReferenceOTU7	D_0__Eukaryota; D_1__Opisthokonta; D_2__Holozoa; D_3__Metazoa (Animalia); D_4__Eumetazoa; D_5__Bilateria; D_6__Nematoda; D_7__Chromadorea; D_8__Tylenchida	0.3	0.0	0.7	0.4	0.0	0.0
AY284729.1.1645	D_0__Eukaryota; D_1__Opisthokonta; D_2__Holozoa; D_3__Metazoa (Animalia); D_4__Eumetazoa; D_5__Bilateria; D_6__Nematoda; D_7__Enoplea; D_8__Enoplia; D_9__Triplonchida	0.4	0.3	0.5	0.5	0.0	0.0
CAWI010039461.798.2604	D_0__Eukaryota; D_1__Opisthokonta; D_2__Holozoa; D_3__Metazoa (Animalia); D_4__Eumetazoa; D_5__Bilateria; D_6__Rotifera; D_7__Bdelloidea; D_8__Adinetida; D_9__Adineta vaga	3.5	3.8	3.1	3.9	1.9	0.0
GACQ01020207.292.2090	D_0__Eukaryota; D_1__Opisthokonta; D_2__Holozoa; D_3__Metazoa (Animalia); D_4__Eumetazoa; D_5__Bilateria; D_6__Rotifera; D_7__Monogononta	1.2	1.0	1.5	1.4	0.6	0.0
New.ReferenceOTU700	D_0__Eukaryota; D_1__Opisthokonta; D_2__Holozoa; D_3__Metazoa (Animalia); D_4__Eumetazoa; D_5__Bilateria; D_6__Rotifera; D_7__Monogononta	0.5	0.9	0.0	0.2	2.8	0.0
AJ487048.1.1717	D_0__Eukaryota; D_1__Opisthokonta; D_2__Holozoa; D_3__Metazoa (Animalia); D_4__Eumetazoa; D_5__Bilateria; D_6__Rotifera; D_7__Monogononta	0.2	0.2	0.2	0.2	0.1	0.0
GU070888.1.1815	D_0__Eukaryota; D_1__Opisthokonta; D_2__Holozoa; D_3__Metazoa (Animalia); D_4__Eumetazoa; D_5__Bilateria; D_6__Rotifera; D_7__Monogononta; D_8__Flosculariacea	4.3	2.3	6.4	5.1	0.8	0.0
AF154566.1.1733	D_0__Eukaryota; D_1__Opisthokonta; D_2__Holozoa; D_3__Metazoa (Animalia); D_4__Eumetazoa; D_5__Bilateria; D_6__Rotifera; D_7__Monogononta; D_8__Flosculariacea	1.6	0.7	2.6	1.9	0.1	0.0
New.ReferenceOTU498	D_0__Eukaryota; D_1__Opisthokonta; D_2__Holozoa; D_3__Metazoa (Animalia); D_4__Eumetazoa; D_5__Bilateria; D_6__Rotifera; D_7__Monogononta; D_8__Ploimida	0.6	0.5	0.7	0.5	1.0	0.0
New.ReferenceOTU769	D_0__Eukaryota; D_1__Opisthokonta; D_2__Holozoa; D_3__Metazoa (Animalia); D_4__Eumetazoa; D_5__Cnidaria; D_6__Anthozoa; D_7__Hexacorallia; D_8__Actiniaria; D_9__Nematostella vectensis (starlet sea anemone)	0.2	0.0	0.4	0.2	0.0	0.0
New.ReferenceOTU1137	D_0__Eukaryota; D_1__Opisthokonta; D_2__Nucletmycea; D_3__Discicristoidea; D_4__Nucleariida; D_5__Nucleariidae; D_6__Nuclearia	0.2	0.2	0.2	0.3	0.0	0.0
AF372796.1.1669	D_0__Eukaryota; D_1__Opisthokonta; D_2__Nucletmycea; D_3__Fungi; D_4__Cryptomycota; D_5__LKM11	0.3	0.3	0.3	0.4	0.0	0.0
FJ810604.1.1240	D_0__Eukaryota; D_1__Opisthokonta; D_2__Nucletmycea; D_3__Fungi; D_4__Cryptomycota; D_5__LKM11	0.2	0.1	0.2	0.0	0.1	2.3
AY082996.1.1757	D_0__Eukaryota; D_1__Opisthokonta; D_2__Nucletmycea; D_3__Fungi; D_4__Cryptomycota; D_5__LKM11; D_6__uncultured eukaryote	1.9	2.2	1.6	2.1	1.4	0.0
EU278606.1.1737	D_0__Eukaryota; D_1__Opisthokonta; D_2__Nucletmycea; D_3__Fungi; D_4__Dikarya; D_5__Ascomycota; D_6__Pezizomycotina; D_7__Eurotiomycetes; D_8__Eurotiales; D_9__Trichocomaceae; D_10__Penicillium	0.3	0.4	0.2	0.2	0.3	2.4
EU978452.1.1702	D_0__Eukaryota; D_1__Opisthokonta; D_2__Nucletmycea; D_3__Fungi; D_4__Dikarya; D_5__Basidiomycota; D_6__Pucciniomycotina; D_7__Cystobasidiomycetes; D_8__Cystobasidiales; D_9__Cystobasidiaceae	0.9	0.2	1.7	0.0	0.0	16.8
AF255360.1.1665	D_0__Eukaryota; D_1__SAR; D_2__Alveolata; D_3__Ciliophora; D_4__Intramacronucleata; D_5__Conthreep; D_6__Oligohymenophorea; D_7__Peniculia; D_8__Paramecium; D_9__Paramecium putrinum	0.4	0.7	0.0	0.5	0.0	0.0
KJ925235.1.1651	D_0__Eukaryota; D_1__SAR; D_2__Alveolata; D_3__Ciliophora; D_4__Intramacronucleata; D_5__Conthreep; D_6__Prostomatea; D_7__Cryptocaryon; D_8__uncultured eukaryote	1.4	2.6	0.0	0.5	8.1	0.0
KJ925254.1.1590	D_0__Eukaryota; D_1__SAR; D_2__Alveolata; D_3__Ciliophora; D_4__Intramacronucleata; D_5__Litostomatea; D_6__Haptoria; D_7__Pelagodileptus	0.2	0.4	0.0	0.2	0.3	0.0
New.ReferenceOTU80	D_0__Eukaryota; D_1__SAR; D_2__Alveolata; D_3__Dinoflagellata; D_4__Dinophyceae	1.0	0.5	1.4	1.2	0.1	0.0
FJ000203.1.1378	D_0__Eukaryota; D_1__SAR; D_2__Alveolata; D_3__Dinoflagellata; D_4__Dinophyceae; D_5__Gymnodiniphycidae; D_6__Gyrodinium; D_7__uncultured eukaryote	0.2	0.2	0.2	0.3	0.0	0.0
New.ReferenceOTU20	D_0__Eukaryota; D_1__SAR; D_2__Alveolata; D_3__Dinoflagellata; D_4__Dinophyceae; D_5__Gymnodiniphycidae; D_6__Suessiaceae; D_7__Biecheleria	2.3	1.9	2.7	2.5	1.5	0.0
New.ReferenceOTU1075	D_0__Eukaryota; D_1__SAR; D_2__Alveolata; D_3__Dinoflagellata; D_4__Dinophyceae; D_5__Gymnodiniphycidae; D_6__Suessiaceae; D_7__Biecheleria	1.4	1.3	1.5	1.7	0.0	0.0
AB686253.1.1713	D_0__Eukaryota; D_1__SAR; D_2__Alveolata; D_3__Dinoflagellata; D_4__Dinophyceae; D_5__Gymnodiniphycidae; D_6__Suessiaceae; D_7__Biecheleria	0.3	0.2	0.3	0.3	0.0	0.0
New.ReferenceOTU1050	D_0__Eukaryota; D_1__SAR; D_2__Alveolata; D_3__Dinoflagellata; D_4__Dinophyceae; D_5__Peridiniphycidae; D_6__Thoracosphaeraceae; D_7__uncultured	0.4	0.3	0.5	0.4	0.0	0.0
New.ReferenceOTU554	D_0__Eukaryota; D_1__SAR; D_2__Stramenopiles; D_3__MAST-12; D_4__MAST-12C	0.7	0.8	0.6	0.6	1.7	0.0
AF396684.1.1780	D_0__Eukaryota; D_1__SAR; D_2__Stramenopiles; D_3__Peronosporomycetes; D_4__Aphanomyces	2.3	1.6	3.1	2.8	0.0	0.0
AY919745.1.1742	D_0__Eukaryota; D_1__SAR; D_2__Stramenopiles; D_3__Peronosporomycetes; D_4__Saprolegnia	0.2	0.1	0.2	0.2	0.0	0.0
New.ReferenceOTU451	Unassigned	1.3	2.0	0.5	0.5	3.0	9.0
New.ReferenceOTU656	Unassigned	0.3	0.0	0.5	0.3	0.0	0.0
New.ReferenceOTU670	Unassigned	0.2	0.3	0.0	0.0	0.4	1.4
New.ReferenceOTU135	Unassigned	0.2	0.3	0.0	0.1	0.7	0.0

Samples (%ALL, average abundance from 55 samples) grouped by template (DNA/RNA) and water system properties (cold water/hot water/biofilm).

Table S7. TOP 50 Operational taxonomic units (OTUs) in DWDS D.

OTU ID	Taxonomic classification	% ALL	%DNA	%RNA	%cold water	%hot water	%biofilms
KC188996.1.1829	D_0__Eukaryota	3.6	1.2	6.4	0.1	0.0	28.6
KJ925353.1.1495	D_0__Eukaryota	1.3	1.7	0.9	1.6	1.2	0.0
New.ReferenceOTU807	D_0__Eukaryota; D_1__Amoebozoa; D_2__Discosea; D_3__Flabellinia; D_4__Vannellida; D_5__uncultured; D_6__uncultured Amoebozoa	0.4	0.2	0.6	0.3	0.5	0.4
EF024971.1.2014	D_0__Eukaryota; D_1__Amoebozoa; D_2__LEMD255; D_3__uncultured Eimeriidae	1.2	0.9	1.6	1.7	0.1	0.0
AY919786.1.1863	D_0__Eukaryota; D_1__Amoebozoa; D_2__LKM74	2.3	1.3	3.5	2.6	1.6	1.5
EU349232.1.1720	D_0__Eukaryota; D_1__Incertae Sedis; D_2__Ancyromonadida; D_3__Ancyromonas	2.4	1.9	3.0	3.3	0.2	0.1
New.ReferenceOTU1090	D_0__Eukaryota; D_1__Opisthokonta	1.0	0.0	2.2	1.4	0.0	0.0
New.ReferenceOTU51	D_0__Eukaryota; D_1__Opisthokonta	0.4	0.5	0.2	0.5	0.0	0.0
FJ577820.1.1699	D_0__Eukaryota; D_1__Opisthokonta; D_2__Holozoa; D_3__Metazoa (Animalia); D_4__Eumetazoa; D_5__Bilateria; D_6__Annelida; D_7__Clitellata; D_8__Oligochaeta; D_9__Haplotaxida	0.3	0.4	0.2	0.3	0.5	0.1
ABRN02315288.22.1468	D_0__Eukaryota; D_1__Opisthokonta; D_2__Holozoa; D_3__Metazoa (Animalia); D_4__Eumetazoa; D_5__Bilateria; D_6__Chordata; D_7__Vertebrata; D_8__Gnathostomata; D_9__Euteleostomi; D_10__Tetrapoda; D_11__Mammalia	0.5	0.4	0.7	0.0	3.2	0.2
JN185462.1.1710	D_0__Eukaryota; D_1__Opisthokonta; D_2__Holozoa; D_3__Metazoa (Animalia); D_4__Eumetazoa; D_5__Bilateria; D_6__Gastrotricha; D_7__Chaetonotida	0.3	0.6	0.0	0.3	0.5	0.0
New.ReferenceOTU365	D_0__Eukaryota; D_1__Opisthokonta; D_2__Holozoa; D_3__Metazoa (Animalia); D_4__Eumetazoa; D_5__Bilateria; D_6__Hemichordata; D_7__Enteropneusta; D_8__Harrimaniidae	0.3	0.1	0.6	0.3	0.2	0.3
KJ636252.1.1690	D_0__Eukaryota; D_1__Opisthokonta; D_2__Holozoa; D_3__Metazoa (Animalia); D_4__Eumetazoa; D_5__Bilateria; D_6__Nematoda; D_7__Chromadorea; D_8__Monhysterida	1.4	1.7	1.0	1.8	0.7	0.0
New.ReferenceOTU179	D_0__Eukaryota; D_1__Opisthokonta; D_2__Holozoa; D_3__Metazoa (Animalia); D_4__Eumetazoa; D_5__Bilateria; D_6__Nematoda; D_7__Chromadorea; D_8__Monhysterida	0.8	1.3	0.1	0.8	1.3	0.0
AY284692.1.1601	D_0__Eukaryota; D_1__Opisthokonta; D_2__Holozoa; D_3__Metazoa (Animalia); D_4__Eumetazoa; D_5__Bilateria; D_6__Nematoda; D_7__Chromadorea; D_8__Monhysterida	0.5	1.0	0.0	0.7	0.0	0.0
New.ReferenceOTU1140	D_0__Eukaryota; D_1__Opisthokonta; D_2__Holozoa; D_3__Metazoa (Animalia); D_4__Eumetazoa; D_5__Bilateria; D_6__Nematoda; D_7__Chromadorea; D_8__Monhysterida	0.4	0.7	0.0	0.5	0.1	0.0
New.ReferenceOTU692	D_0__Eukaryota; D_1__Opisthokonta; D_2__Holozoa; D_3__Metazoa (Animalia); D_4__Eumetazoa; D_5__Bilateria; D_6__Nematoda; D_7__Chromadorea; D_8__Monhysterida	0.3	0.6	0.0	0.4	0.2	0.0
AY284729.1.1645	D_0__Eukaryota; D_1__Opisthokonta; D_2__Holozoa; D_3__Metazoa (Animalia); D_4__Eumetazoa; D_5__Bilateria; D_6__Nematoda; D_7__Enoplea; D_8__Enoplia; D_9__Triplonchida	0.6	0.9	0.2	0.0	0.0	4.7
CAWI010039461.798.2604	D_0__Eukaryota; D_1__Opisthokonta; D_2__Holozoa; D_3__Metazoa (Animalia); D_4__Eumetazoa; D_5__Bilateria; D_6__Rotifera; D_7__Bdelloidea; D_8__Adinetida; D_9__Adineta vaga	0.5	0.4	0.6	0.6	0.3	0.0
GACQ01020207.292.2090	D_0__Eukaryota; D_1__Opisthokonta; D_2__Holozoa; D_3__Metazoa (Animalia); D_4__Eumetazoa; D_5__Bilateria; D_6__Rotifera; D_7__Monogononta	0.3	0.1	0.6	0.5	0.0	0.0
AB753790.1.1745	D_0__Eukaryota; D_1__Opisthokonta; D_2__Holozoa; D_3__Metazoa (Animalia); D_4__Eumetazoa; D_5__Bilateria; D_6__Tardigrada; D_7__Eutardigrada; D_8__Parachela	0.7	1.3	0.0	0.5	2.0	0.1
New.ReferenceOTU577	D_0__Eukaryota; D_1__Opisthokonta; D_2__Holozoa; D_3__Metazoa (Animalia); D_4__Eumetazoa; D_5__Cnidaria; D_6__Hydrozoa; D_7__Hydroidolina; D_8__Leptothecata; D_9__Gonothyraea loveni	0.6	0.0	1.2	0.8	0.1	0.0
HM628685.1.1543	D_0__Eukaryota; D_1__Opisthokonta; D_2__Nucletmycea; D_3__Fungi; D_4__Cryptomycota; D_5__LKM11	6.5	8.9	3.6	6.0	13.6	0.5
FJ577832.1.1640	D_0__Eukaryota; D_1__Opisthokonta; D_2__Nucletmycea; D_3__Fungi; D_4__Cryptomycota; D_5__LKM11; D_6__uncultured eukaryote	9.7	11.4	7.6	10.5	11.8	2.0
HM487048.1.1346	D_0__Eukaryota; D_1__Opisthokonta; D_2__Nucletmycea; D_3__Fungi; D_4__Cryptomycota; D_5__LKM11; D_6__uncultured fungus	4.6	6.0	3.0	3.6	5.4	9.2
New.ReferenceOTU749	D_0__Eukaryota; D_1__Opisthokonta; D_2__Nucletmycea; D_3__Fungi; D_4__Cryptomycota; D_5__LKM11; D_6__uncultured soil fungus	0.4	0.5	0.3	0.5	0.0	0.0
New.ReferenceOTU264	D_0__Eukaryota; D_1__Opisthokonta; D_2__Nucletmycea; D_3__Fungi; D_4__Dikarya; D_5__Ascomycota; D_6__Pezizomycotina; D_7__Leotiomycetes; D_8__Helotiales	1.1	0.9	1.4	1.3	1.3	0.1
JN705505.1.1364	D_0__Eukaryota; D_1__SAR; D_2__Alveolata; D_3__Ciliophora; D_4__Intramacronucleata; D_5__Conthreep; D_6__Oligohymenophorea; D_7__CV1-2A-17	1.2	1.1	1.3	1.6	0.4	0.2
New.ReferenceOTU183	D_0__Eukaryota; D_1__SAR; D_2__Alveolata; D_3__Ciliophora; D_4__Intramacronucleata; D_5__Conthreep; D_6__Phyllopharyngea; D_7__Cyrtophoria; D_8__Trochilioides; D_9__uncultured eukaryote	1.5	1.1	1.9	1.9	0.6	0.1
New.ReferenceOTU941	D_0__Eukaryota; D_1__SAR; D_2__Alveolata; D_3__Ciliophora; D_4__Intramacronucleata; D_5__Litostomatea; D_6__Haptoria	0.3	0.2	0.5	0.4	0.5	0.0
KJ680554.1.1538	D_0__Eukaryota; D_1__SAR; D_2__Alveolata; D_3__Ciliophora; D_4__Intramacronucleata; D_5__Litostomatea; D_6__Haptoria	0.3	0.2	0.5	0.4	0.0	0.0
FJ577826.1.1617	D_0__Eukaryota; D_1__SAR; D_2__Alveolata; D_3__Ciliophora; D_4__Intramacronucleata; D_5__Spirotrichea; D_6__Euplotia; D_7__Aspidisca; D_8__uncultured ciliate	0.7	1.2	0.1	0.9	0.2	0.0
HM161745.1.1787	D_0__Eukaryota; D_1__SAR; D_2__Stramenopiles; D_3__Ochrophyta; D_4__Chrysophyceae; D_5__Chromulinales	2.0	3.4	0.4	2.8	0.0	0.0
AB520724.1.1665	D_0__Eukaryota; D_1__SAR; D_2__Stramenopiles; D_3__Ochrophyta; D_4__Chrysophyceae; D_5__Chromulinales; D_6__Spumella	15.9	16.5	15.3	16.8	24.6	0.1
New.ReferenceOTU451	Unassigned	4.0	5.4	2.5	0.0	0.2	32.3
New.ReferenceOTU656	Unassigned	3.4	0.8	6.5	4.5	1.1	0.2
New.ReferenceOTU825	Unassigned	2.5	2.3	2.7	3.3	0.2	0.2
New.ReferenceOTU1032	Unassigned	1.5	2.8	0.0	1.6	2.1	0.2
New.ReferenceOTU302	Unassigned	1.1	0.7	1.7	0.9	0.2	3.7
New.ReferenceOTU88	Unassigned	0.9	0.2	1.7	1.2	0.3	0.1
New.ReferenceOTU524	Unassigned	0.7	0.6	0.8	0.9	0.0	0.0
New.ReferenceOTU768	Unassigned	0.6	0.1	1.3	0.7	0.4	0.4
New.ReferenceOTU670	Unassigned	0.6	0.8	0.4	0.0	0.0	4.9
New.ReferenceOTU823	Unassigned	0.5	0.1	1.1	0.3	1.9	0.0
New.ReferenceOTU1033	Unassigned	0.5	0.8	0.2	0.5	0.9	0.0
New.ReferenceOTU454	Unassigned	0.4	0.2	0.7	0.6	0.0	0.0
New.ReferenceOTU832	Unassigned	0.4	0.1	0.6	0.0	0.0	2.6
New.ReferenceOTU187	Unassigned	0.3	0.1	0.6	0.3	0.2	0.7
New.ReferenceOTU521	Unassigned	0.3	0.0	0.7	0.4	0.0	0.0
New.ReferenceOTU1060	Unassigned	0.3	0.1	0.5	0.2	0.9	0.0

Samples (%ALL, average abundance from 65 samples) grouped by template (DNA/RNA) and water system properties (cold water/hot water/biofilm).

Table S8. TOP 50 Operational taxonomic units (OTUs) in DWDS E.

OTU ID	Taxonomic classification	% ALL	%DNA	%RNA	%cold water	%hot water	%biofilms
KJ925353.1.1495	D_0__Eukaryota	0.5	1.1	0.2	0.6	0.0	0.2
EF032800.1.1760	D_0__Eukaryota; D_1__Amoebozoa; D_2__Discosea; D_3__Flabellinia; D_4__Vannellida; D_5__Vannella	0.5	0.0	0.7	0.6	0.0	0.0
New.ReferenceOTU156	D_0__Eukaryota; D_1__Amoebozoa; D_2__Discosea; D_3__Flabellinia; D_4__Vannellida; D_5__Vannella	0.4	0.0	0.5	0.5	0.0	0.0
EF024971.1.2014	D_0__Eukaryota; D_1__Amoebozoa; D_2__LEMD255; D_3__uncultured Eimeriidae	0.8	0.3	1.0	0.9	0.0	0.5
AY919786.1.1863	D_0__Eukaryota; D_1__Amoebozoa; D_2__LKM74	0.4	0.2	0.4	0.3	0.0	1.2
JF694280.1.1946	D_0__Eukaryota; D_1__Amoebozoa; D_2__Tubulinea; D_3__Arcellinida; D_4__Phryganellina; D_5__Cryptodifflugia; D_6__Cryptodifflugia operculata	0.7	0.1	0.9	0.9	0.0	0.0
New.ReferenceOTU471	D_0__Eukaryota; D_1__Amoebozoa; D_2__Tubulinea; D_3__Arcellinida; D_4__Phryganellina; D_5__Cryptodifflugia; D_6__Cryptodifflugia operculata	0.7	0.0	0.9	0.8	0.0	0.0
New.ReferenceOTU67	D_0__Eukaryota; D_1__Amoebozoa; D_2__Tubulinea; D_3__Euamoebida	3.2	0.8	4.1	2.9	0.0	6.4
JX159170.1.1809	D_0__Eukaryota; D_1__Archaeplastida; D_2__Chloroplastida; D_3__Charophyta; D_4__Phragmoplastophyta; D_5__Streptophyta; D_6__Embryophyta; D_7__Tracheophyta; D_8__Spermatophyta; D_9__Magnoliophyta	0.4	0.0	0.6	0.5	0.0	0.0
New.ReferenceOTU649	D_0__Eukaryota; D_1__Archaeplastida; D_2__Chloroplastida; D_3__Charophyta; D_4__Phragmoplastophyta; D_5__Streptophyta; D_6__Embryophyta; D_7__Tracheophyta; D_8__Spermatophyta; D_9__Magnoliophyta; D_10__Fabales; D_11__Phaseolus; D_12__Phaseolus acutifolius (tepary bean)	0.7	0.1	0.9	0.0	0.0	4.4
New.ReferenceOTU486	D_0__Eukaryota; D_1__Opisthokonta	1.1	0.1	1.5	1.2	0.0	0.9
AZAQ01001705.16275.180	D_0__Eukaryota; D_1__Opisthokonta; D_2__Holozoa; D_3__Metazoa (Animalia); D_4__Eumetazoa; D_5__Bilateria; D_6__Arthropoda; D_7__Chelicerata; D_8__Arachnida; D_9__Araneae	1.7	0.0	2.3	2.2	0.0	0.0
New.ReferenceOTU1146	D_0__Eukaryota; D_1__Opisthokonta; D_2__Holozoa; D_3__Metazoa (Animalia); D_4__Eumetazoa; D_5__Bilateria; D_6__Arthropoda; D_7__Crustacea; D_8__Maxillopoda; D_9__Copepoda	1.1	3.1	0.3	0.1	0.0	6.7
ABRN02315288.22.1468	D_0__Eukaryota; D_1__Opisthokonta; D_2__Holozoa; D_3__Metazoa (Animalia); D_4__Eumetazoa; D_5__Bilateria; D_6__Chordata; D_7__Vertebrata; D_8__Gnathostomata; D_9__Euteleostomi; D_10__Tetrapoda; D_11__Mammalia	1.6	2.2	1.3	1.5	5.0	0.2
New.ReferenceOTU333	D_0__Eukaryota; D_1__Opisthokonta; D_2__Holozoa; D_3__Metazoa (Animalia); D_4__Eumetazoa; D_5__Bilateria; D_6__Nematoda; D_7__Chromadorea	1.1	3.0	0.4	1.4	0.0	0.0
AJ875123.1.1729	D_0__Eukaryota; D_1__Opisthokonta; D_2__Holozoa; D_3__Metazoa (Animalia); D_4__Eumetazoa; D_5__Bilateria; D_6__Nematoda; D_7__Chromadorea; D_8__Araeolaimida	8.8	16.2	6.0	8.4	33.4	0.8
KJ636252.1.1690	D_0__Eukaryota; D_1__Opisthokonta; D_2__Holozoa; D_3__Metazoa (Animalia); D_4__Eumetazoa; D_5__Bilateria; D_6__Nematoda; D_7__Chromadorea; D_8__Monhysterida	10.4	4.2	12.7	12.8	0.0	2.3
New.ReferenceOTU1140	D_0__Eukaryota; D_1__Opisthokonta; D_2__Holozoa; D_3__Metazoa (Animalia); D_4__Eumetazoa; D_5__Bilateria; D_6__Nematoda; D_7__Chromadorea; D_8__Monhysterida	1.5	3.2	0.9	1.9	0.0	0.2
AY284692.1.1601	D_0__Eukaryota; D_1__Opisthokonta; D_2__Holozoa; D_3__Metazoa (Animalia); D_4__Eumetazoa; D_5__Bilateria; D_6__Nematoda; D_7__Chromadorea; D_8__Monhysterida	0.7	1.2	0.6	0.8	0.0	0.6
FJ040408.1.1660	D_0__Eukaryota; D_1__Opisthokonta; D_2__Holozoa; D_3__Metazoa (Animalia); D_4__Eumetazoa; D_5__Bilateria; D_6__Nematoda; D_7__Chromadorea; D_8__Tylenchida	2.6	3.6	2.3	3.3	0.0	0.0
KF561100.1.1726	D_0__Eukaryota; D_1__Opisthokonta; D_2__Holozoa; D_3__Metazoa (Animalia); D_4__Eumetazoa; D_5__Bilateria; D_6__Rotifera; D_7__Bdelloidea; D_8__Adinetida; D_9__Adineta vaga	1.5	3.6	0.7	0.0	0.0	9.9
GACQ01020207.292.2090	D_0__Eukaryota; D_1__Opisthokonta; D_2__Holozoa; D_3__Metazoa (Animalia); D_4__Eumetazoa; D_5__Bilateria; D_6__Rotifera; D_7__Monogononta	1.7	0.2	2.2	2.0	0.0	0.4
GU070888.1.1815	D_0__Eukaryota; D_1__Opisthokonta; D_2__Holozoa; D_3__Metazoa (Animalia); D_4__Eumetazoa; D_5__Bilateria; D_6__Rotifera; D_7__Monogononta; D_8__Flosculariacea	3.7	0.2	5.0	4.6	0.0	0.7
New.ReferenceOTU590	D_0__Eukaryota; D_1__Opisthokonta; D_2__Holozoa; D_3__Metazoa (Animalia); D_4__Eumetazoa; D_5__Bilateria; D_6__Rotifera; D_7__Monogononta; D_8__Ploimida	0.4	0.0	0.5	0.5	0.0	0.0
New.ReferenceOTU769	D_0__Eukaryota; D_1__Opisthokonta; D_2__Holozoa; D_3__Metazoa (Animalia); D_4__Eumetazoa; D_5__Cnidaria; D_6__Anthozoa; D_7__Hexacorallia; D_8__Actiniaria; D_9__Nematostella vectensis (starlet sea anemone)	0.5	0.1	0.7	0.7	0.0	0.0
DQ536488.1.1799	D_0__Eukaryota; D_1__Opisthokonta; D_2__Nucletmycea; D_3__Fungi; D_4__Chytridiomycota; D_5__Incertae Sedis; D_6__Chytridiomycetes; D_7__Rhizophydium sp. MP8	1.4	1.1	1.5	1.8	0.0	0.0
New.ReferenceOTU68	D_0__Eukaryota; D_1__Opisthokonta; D_2__Nucletmycea; D_3__Fungi; D_4__Cryptomycota; D_5__LKM11	0.7	2.0	0.2	0.0	0.0	4.4
HM628685.1.1543	D_0__Eukaryota; D_1__Opisthokonta; D_2__Nucletmycea; D_3__Fungi; D_4__Cryptomycota; D_5__LKM11	0.6	0.4	0.7	0.8	0.0	0.1
EU091865.1.1417	D_0__Eukaryota; D_1__Opisthokonta; D_2__Nucletmycea; D_3__Fungi; D_4__Cryptomycota; D_5__LKM11	0.4	0.6	0.4	0.6	0.0	0.0
FJ577832.1.1640	D_0__Eukaryota; D_1__Opisthokonta; D_2__Nucletmycea; D_3__Fungi; D_4__Cryptomycota; D_5__LKM11; D_6__uncultured eukaryote	1.4	0.5	1.7	1.7	0.0	0.0
EU278606.1.1737	D_0__Eukaryota; D_1__Opisthokonta; D_2__Nucletmycea; D_3__Fungi; D_4__Dikarya; D_5__Ascomycota; D_6__Pezizomycotina; D_7__Eurotiomycetes; D_8__Eurotiales; D_9__Trichocomaceae; D_10__Penicillium	4.1	3.9	4.2	0.8	56.6	0.1
New.ReferenceOTU235	D_0__Eukaryota; D_1__Opisthokonta; D_2__Nucletmycea; D_3__LKM15; D_4__uncultured eukaryote	1.3	0.3	1.7	1.6	0.0	0.0
EF032797.1.1710	D_0__Eukaryota; D_1__SAR; D_2__Alveolata; D_3__Ciliophora; D_4__Intramacronucleata; D_5__Conthreep; D_6__Colpodea; D_7__Cyrtolophosidida	0.6	0.3	0.6	0.7	0.0	0.0
New.ReferenceOTU1063	D_0__Eukaryota; D_1__SAR; D_2__Alveolata; D_3__Ciliophora; D_4__Intramacronucleata; D_5__Conthreep; D_6__Nassophorea	1.0	0.3	1.2	1.2	0.0	0.0
Z22879.1.1718	D_0__Eukaryota; D_1__SAR; D_2__Alveolata; D_3__Ciliophora; D_4__Intramacronucleata; D_5__Conthreep; D_6__Oligohymenophorea; D_7__Scuticociliatia; D_8__Cyclidium	1.5	0.7	1.8	1.6	0.0	1.5
HM030917.1.1568	D_0__Eukaryota; D_1__SAR; D_2__Alveolata; D_3__Ciliophora; D_4__Intramacronucleata; D_5__Conthreep; D_6__Phyllopharyngea; D_7__Cyrtophoria; D_8__Chilodonella; D_9__Chilodonella uncinata	0.4	0.2	0.5	0.5	0.0	0.0
AY642746.1.1211	D_0__Eukaryota; D_1__SAR; D_2__Stramenopiles; D_3__Ochrophyta; D_4__Chrysophyceae	0.6	0.0	0.8	0.7	0.0	0.1
AB520724.1.1665	D_0__Eukaryota; D_1__SAR; D_2__Stramenopiles; D_3__Ochrophyta; D_4__Chrysophyceae; D_5__Chromulinales; D_6__Spumella	2.9	7.7	1.0	3.6	0.0	0.0
HQ643757.1.1793	D_0__Eukaryota; D_1__SAR; D_2__Stramenopiles; D_3__Peronosporomycetes; D_4__Pythium	1.1	1.4	1.0	0.2	0.0	6.5
New.ReferenceOTU187	Unassigned	2.2	0.1	3.0	2.8	0.0	0.0
New.ReferenceOTU681	Unassigned	1.4	0.6	1.6	1.7	0.0	0.0
New.ReferenceOTU137	Unassigned	1.3	2.1	1.0	1.6	0.2	0.1
New.ReferenceOTU1041	Unassigned	1.3	0.1	1.8	1.5	0.0	1.1
New.ReferenceOTU1024	Unassigned	1.0	1.5	0.8	0.1	0.0	6.4
New.ReferenceOTU165	Unassigned	0.9	0.3	1.1	1.1	0.0	0.0
New.ReferenceOTU88	Unassigned	0.7	0.3	0.9	0.9	0.0	0.1
New.ReferenceOTU182	Unassigned	0.7	0.0	1.0	0.0	0.0	4.5
New.ReferenceOTU859	Unassigned	0.7	0.4	0.8	0.9	0.0	0.0
New.ReferenceOTU704	Unassigned	0.6	0.0	0.8	0.7	0.0	0.0
New.ReferenceOTU1011	Unassigned	0.4	0.0	0.5	0.4	0.0	0.2

Samples (%ALL, average abundance from 33 samples) grouped by template (DNA/RNA) and water system properties (cold water/hot water/biofilm).
