# Supplementary material for: Active eukaryotes in drinking water distribution systems of ground and surface waterworks
Source: Microbiome. 2019 Jul 3;7:99. doi: 10.1186/s40168-019-0715-5 (PMC6610866; doi:10.1186/s40168-019-0715-5)
Supplement: Supplementary file 6 — Boxplot of alpha-diversity Shannon values grouped by cold water, hot water, and biofilms in each DWDSs A–E (DNA + RNA) (Figure S2). Boxplot of alpha-diversity Shannon values in cold water (Figure S3). (PDF 255 kb) [file 40168_2019_715_MOESM6_ESM.pdf]

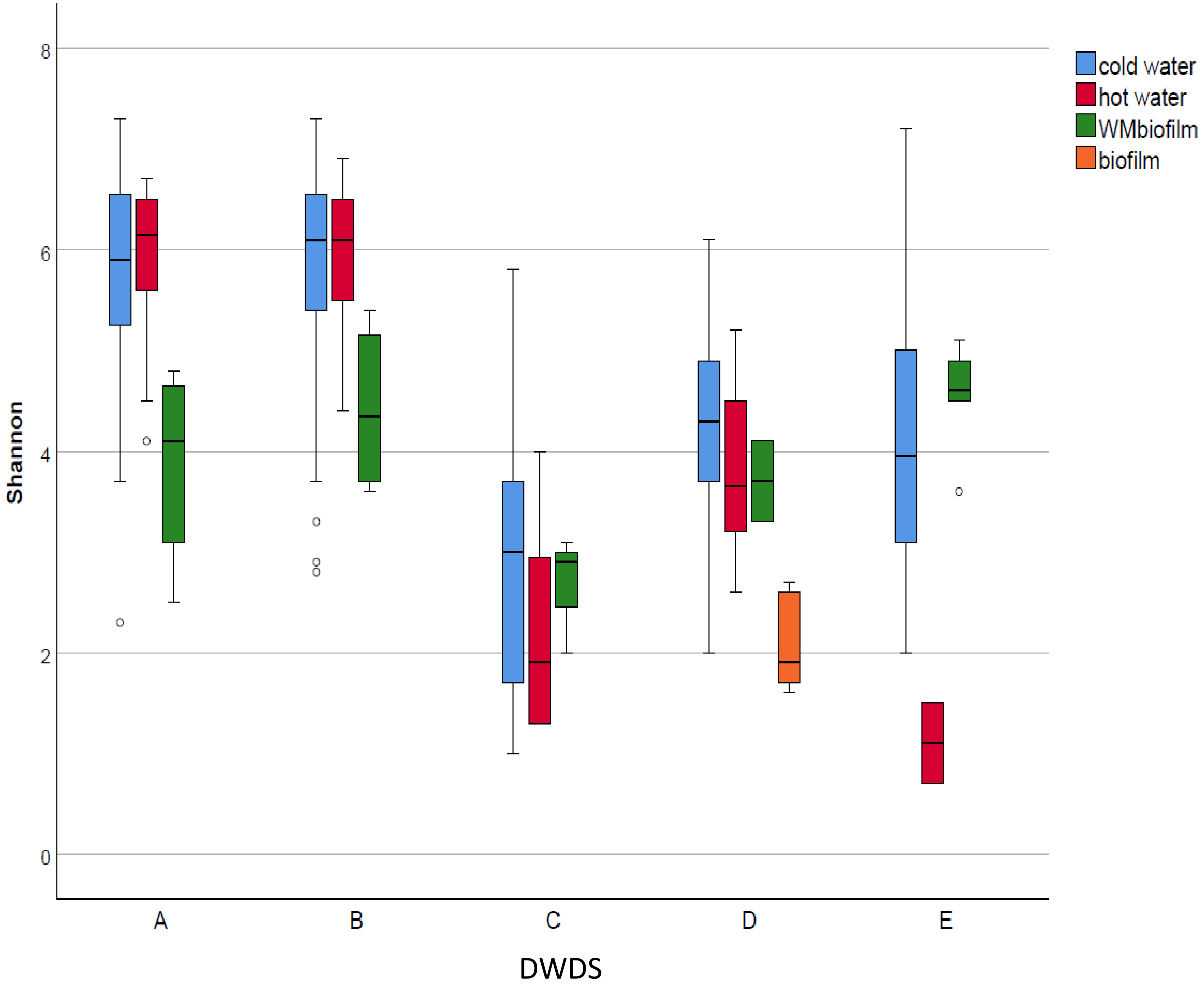

Fig. S2. Boxplot of alpha-diversity Shannon values grouped by cold water, hot water and biofilms in each DWDS A–E (DNA + RNA). WMbiofilm = water meter biofilm, biofilm = pipeline biofilm.

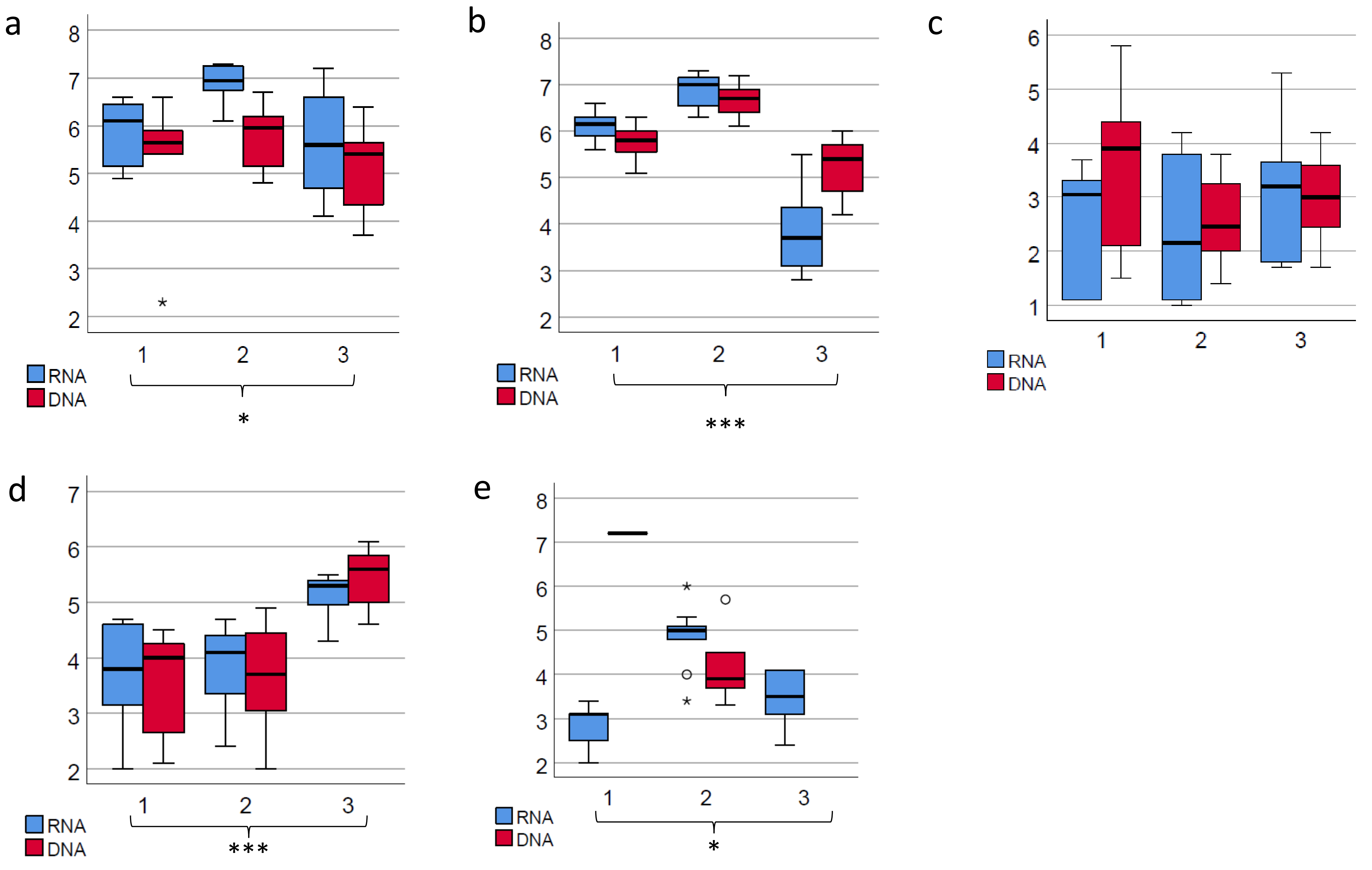

Fig. S3. Boxplot of alpha-diversity Shannon values in cold water. a–b) non-disinfected cold water in DWDSs A–B and c–e) disinfected cold water in DWDSs C–E at different locations grouped by RNA/DNA. Statistical significance of the location: * P < 0.05; ** P < 0.01; *** P < 0.001.
