# Supplementary material for: Active eukaryotes in drinking water distribution systems of ground and surface waterworks
Source: Microbiome. 2019 Jul 3;7:99. doi: 10.1186/s40168-019-0715-5 (PMC6610866; doi:10.1186/s40168-019-0715-5)
Supplement: Supplementary file 7 — Heatmap clustering analysis by activity recovery (%) of 66 Amoebozoa OTUs (Figure S4.), 139 Fungi OTUs (Figure S5.), 110 Metazoa (animal) OTUs (Figure S6.), 84 Chloroplastida OTUs (Figure S7), 234 Alveolata OTUs (Figure S8.), 148 Stramenopiles OTUs (Figure S9.), and 127 Rhizaria OTUs (Figure S10.). Red color represents a high activity recovery, i.e., relative abundance (%) is higher in RNA than DNA; blue color represents a low activity recovery. (PDF 613 kb) [file 40168_2019_715_MOESM7_ESM.pdf]

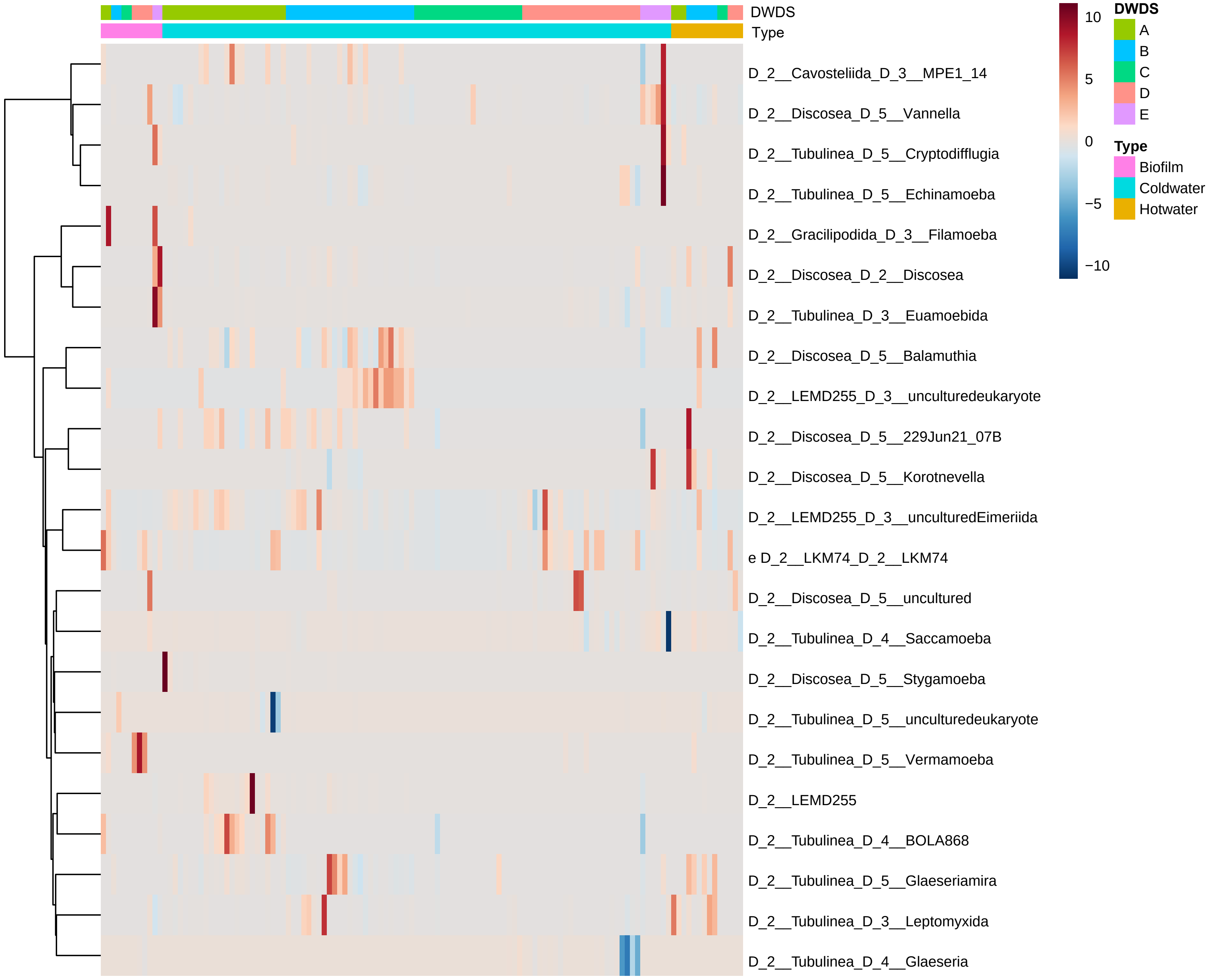

Fig. S4. Heatmap clustering analysis of 66 Amoebozoa OTUs by activity recovery (%).

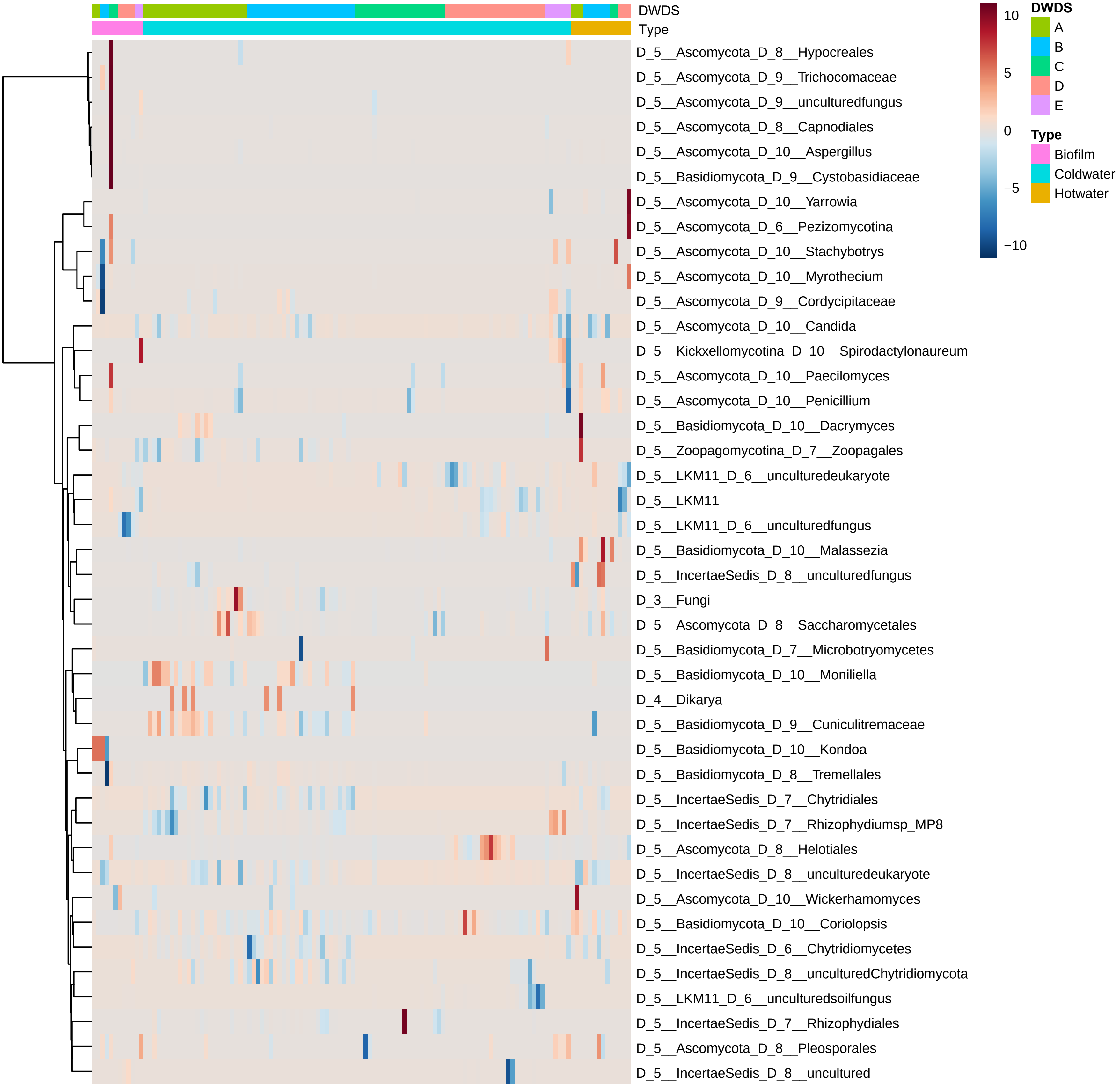

Fig. S5. Heatmap clustering analysis of 139 Fungi OTUs by activity recovery (%).

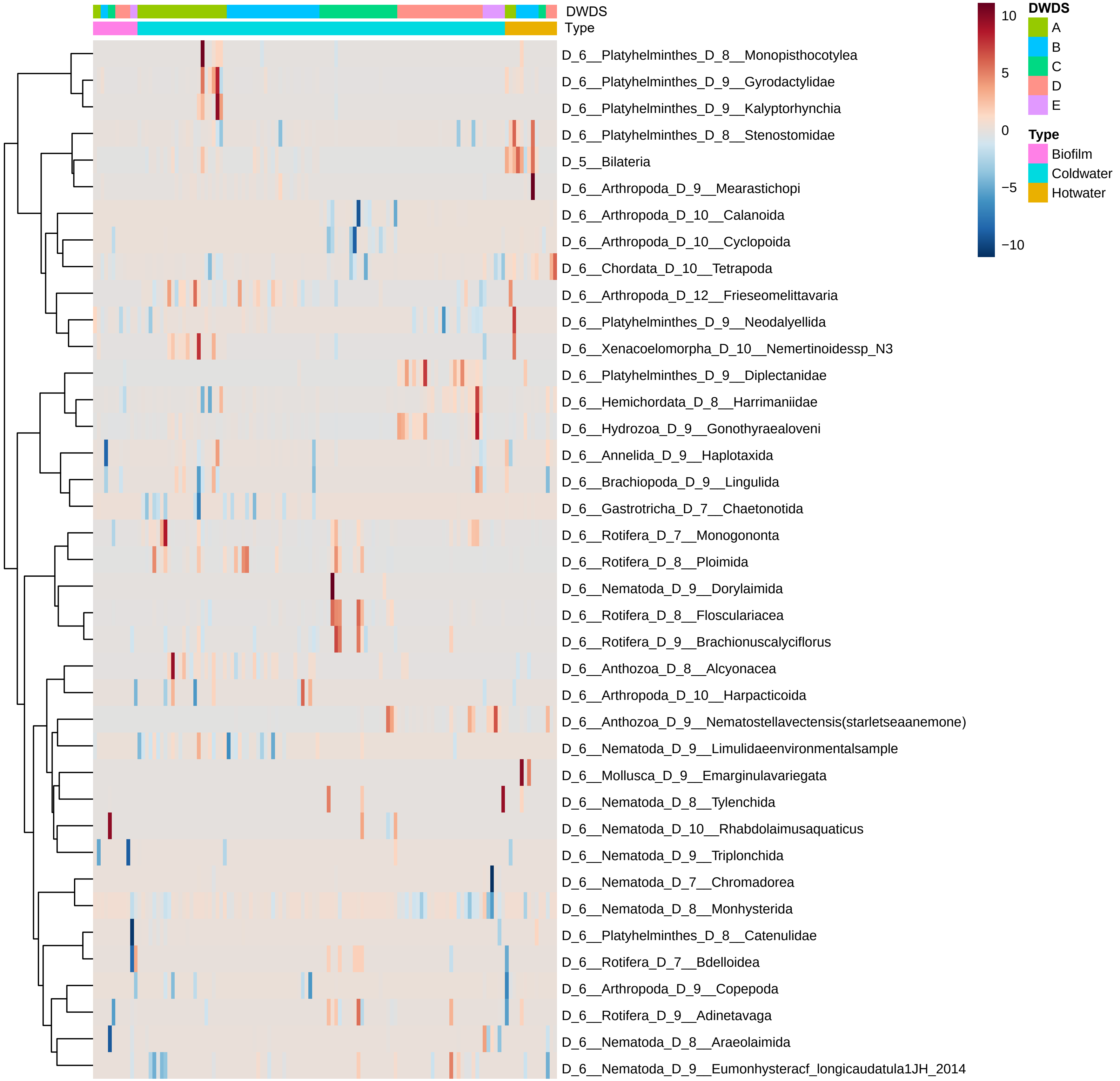

Fig. S6. Heatmap clustering analysis of 110 Metazoa (animal) OTUs by activity recovery (%).

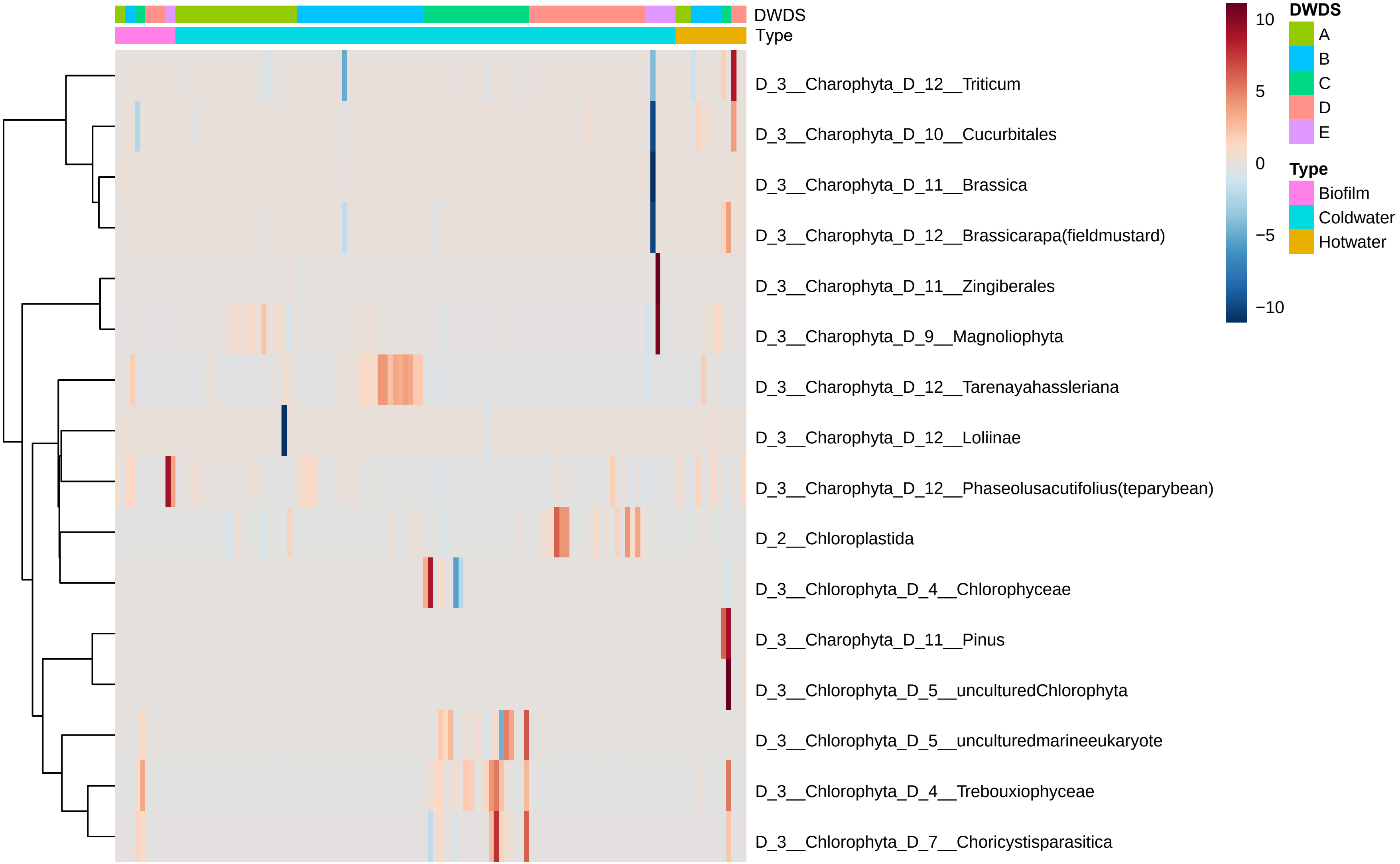

Fig. S7. Heatmap clustering analysis of 84 Chloroplastida OTUs by activity recovery (%).

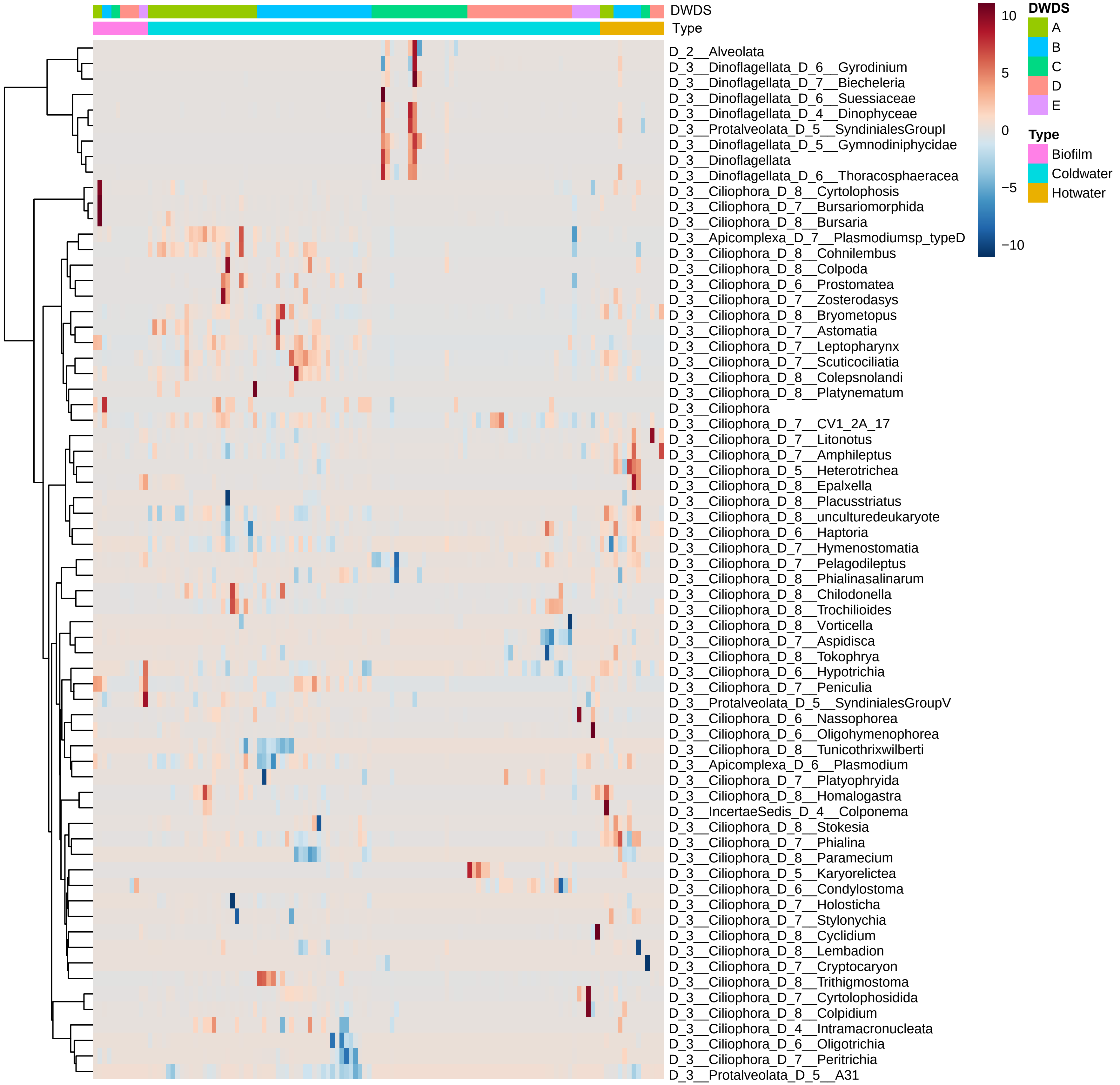

Fig. S8. Heatmap clustering analysis of 234 Alveolata OTUs by activity recovery (%).

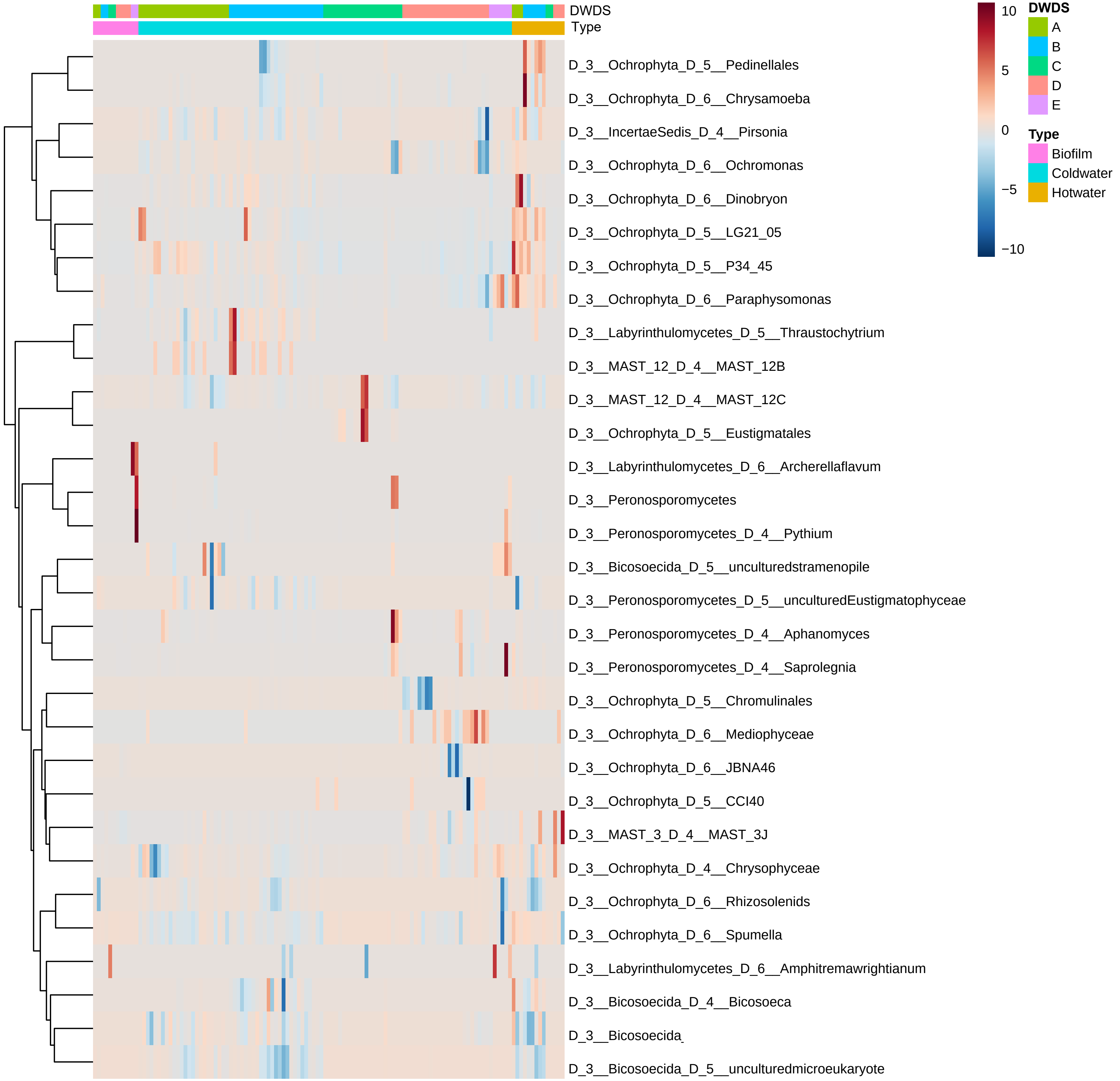

Fig. S9. Heatmap clustering analysis of 148 Stramenopiles OTUs by activity recovery (%).

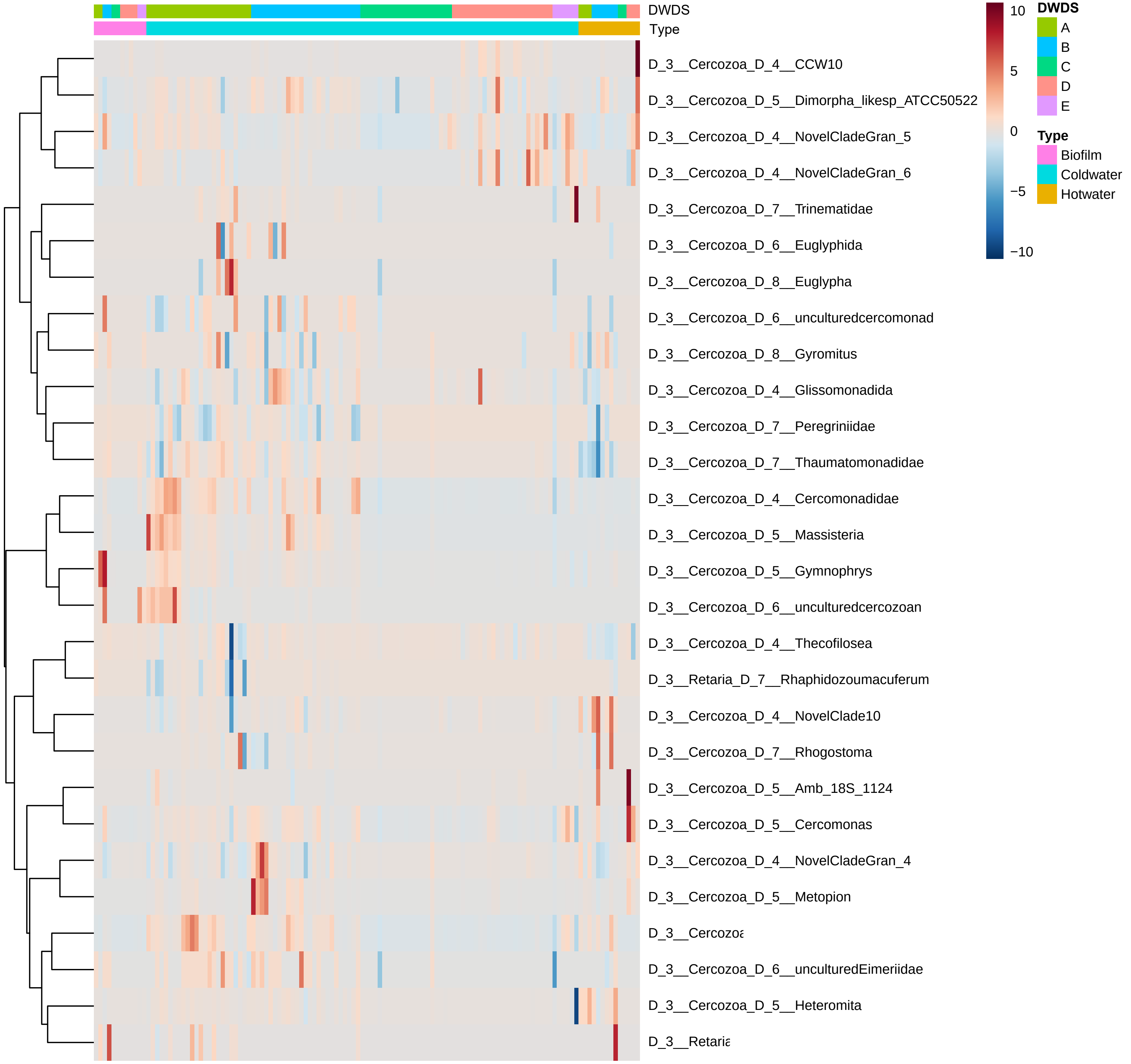

Fig. S10. Heatmap clustering analysis of 127 Rhizaria OTUs by activity recovery (%).
