# Supplementary material for: Active eukaryotes in drinking water distribution systems of ground and surface waterworks
Source: Microbiome. 2019 Jul 3;7:99. doi: 10.1186/s40168-019-0715-5 (PMC6610866; doi:10.1186/s40168-019-0715-5)
Supplement: Supplementary file 8 — Possible pathogenic or opportunistic eukaryotes and free-living amoeba in cold water (Table S9.), hot water (Table S10.), and biofilms (Table S11.) (PDF 271 kb) [file 40168_2019_715_MOESM8_ESM.pdf]

Table S9. Free-living amoeba and potentially pathogenic eukaryotes in cold drinking water.

		Cold water									
		A		B		C		D		E	
		DNA (N = 23)	RNA (N = 24)	DNA (N = 23)	RNA (N = 24)	DNA (N = 22)	RNA (N = 21)	DNA (N = 24)	RNA (N = 23)	DNA (N = 6)	RNA (N = 20)
Free living amoeba	*Balamuthia* (uncultured eukaryote) NCR OTU167741	0–8 (4)	0–42 (11)	0–9 (16)	0–25 (21)	0	0–2 (1)	0	0	0–6 (1)	0
	Vermamoeba vermiformis NR OTU494	0	0–1 (1)	0	0	0–1 (1)	0–1 (1)	0	0–3 (3)	0	0
	Vannella epipetala NR OTU56	0–66 (13)	0–99 (19)	0–34 (19)	0–67 (23)	0–2 (1)	0–12 (2)	0	0	0–32 (2)	0–1403[2] (9)
	Vannella EF032800.1.1760	0	0	0	0	0–7 (4)	0–276[2] (6)	0–27 (10)	0–20 (10)	0–2 (2)	0–236[1-3] (17)
	Vannella NR OTU156	0	0	0	0	0	0	0	0	0–16 (2)	1–404[2-3] (20)
	Vannella epipetala NCR OTU162079	0–11 (11)	0–10 (17)	0–3 (11)	0–8 (13)	0	0–1 (1)	0	0	0–3 (2)	0–100[2] (9)
	Vannella NCR OTU11860	0	0	0	0	0–1(2)	0–17 (1)	0–2 (5)	0	0–4 (1)	0–10 (9)
	Vannella (uncultured eukaryote) NCR OTU186593	0	0	0	0	0	0	0–3 (5)	0	0	0–1 (1)
Fungi	*Aspergillus niger* NR OTU1061	0–8 (2)	0	0–2 (2)	0	0–3 (2)	0	0	0	0–3 (1)	0–11 (5)
	Aspergillus niger JN256017.1.1705	0–2 (1)	0	0	0	0	0	0	0	0–2 (1)	0–1 (2)
	Aspergillus JF719077.1.1233	0	0	0	0	0–1 (1)	0	0	0	0	0
	Aspergillus niger AY256260.1.1229	0	0	0	0	0–1 (1)	0	0	0	0	0–1 (2)
	Candida NR OTU983	0–30 (16)	0–5 (13)	0–64 (16)	0–16 (12)	0–1 (1)	0–7 (1)	0–43 (11)	0–7 (7)	0–50 (5)	0–214 (10)
	Paecilomyces AB015768.1.1686	0–1 (1)	0	0	0	0–2 (3)	0	0	0	0–1 (1)	0–3 (2)
	Stachybotrys chartarum JF797222.1.2154	0	0	0	0	0	0	0	0	0–15 (1)	0–2 (3)
	Alternaria alternata FM178261.1.2018	0	0–1 (1)	0	0	0–0 (0)	0	0–1 (1)	0	0–10 (2)	0–67 (2)
	Penicillium EU278606.1.1737	0–501[3] (6)	0–10 (7)	0–50 (4)	0–3 (4)	0–1 (3)	0–10 (3)	0–7 (2)	0–77 (5)	0–307[2] (3)	0–933[2-3] (9)
	Penicillium (food metagenome) JN624277.1.1430	0–5 (3)	0	0–1 (1)	0	0–362[2] (6)	0	0	0–2 (1)	0–2 (1)	0–8 (3)
	Penicillium AB293968.1.1585	0–3 (2)	0	0–1 (1)	0	0–3 (2)	0	0	0–1 (1)	0	0–3 (4)
	Penicillium AB245443.1.1745	0–1 (1)	0	0	0	0–2 (1)	0	0	0	0	0–1 (1)
	Penicillium KP027014.1.1579	0–1 (1)	0	0	0	0	0–1 (1)	0	0	0–1 (1)	0–2 (2)
	Penicillium AF454063.1.1667	0	0	0	0	0	0	0	0	0–1 (1)	0–1 (1)

Genera not found: POTENTIAL PATHOGENS: *Naegleria, Giardia, Blastocystis, Acanthamoeba, Cryptosporidium, Babesia, Cyclospora, Isospora, Toxoplasma, Entamoeba;* WORMS/CONTAMINATION INDICATORS: *Dientamoeba, Enterobius, Ascaris;* AMOEBA HOSTS: *Acanthamoeba, Vahlkamphia;* FUNGI WITH POSSIBLE HEALTH EFFECTS: *Exophiala, Fusarium, Mucor, Rhizopus, Trichoderma, Chaetomium, Aureobasidium, Bauveria, Botrytis, Cladosporidium, Epicoccum, Purporeocillium, Sarocladium.*

NR = new reference; NCR = new cleanup reference, N = number of samples.

Table values: minimum and maximum reads per sample, number of positive samples in brackets. Upper index = locations (1–3) marked if >100 reads/sample.

Table S10. Free-living amoeba and potentially pathogenic eukaryotes in hot water.

		Hot water									
		A		B		C		D		E	
		DNA (N = 4)	RNA (N = 4)	DNA (N = 8)	RNA (N = 6)	DNA (N = 5)	RNA (N = 2)	DNA (N = 7)	RNA (N = 3)	DNA (N = 1)	RNA (N = 1)
Free living amoeba	*Balamuthia* (uncultured eukaryote) NCR OTU167741	0–12 (1)	0–13 (1)	0	0–18 (2)	0	0	0	0	0	0
	Vermamoeba vermiformis NR OTU494	0	0	0–2 (2)	0–1 (1)	0	0	0	0	0	0
	Vannella epipetala NR OTU56	0–24 (2)	0–37 (2)	0–74 (3)	0–38 (2)	0	0	0	0	0	0
	Vannella EF032800.1.1760	0	0	0	0–1 (1)	0–20 (1)	0	0–6 (2)	0	0	0
	Vannella NR OTU156	0	0	0	0	0	0	0	0	0	0
	Vannella epipetala NCR OTU162079	0–5 (1)	0–7 (2)	0–6 (3)	0–2 (1)	0	0	0	0	0	0
	Vannella NCR OTU11860	0	0	0	0	0–4 (1)	0	0–7 (1)	0	0	0
	Vannella (uncultured eukaryote) NCR OTU186593	0	0	0	0	0	0	0–3 (1)	0	0	0
Fungi	*Aspergillus niger* NR OTU1061	0–15 (1)	0–10 (1)	0–1 (2)	0–1 (2)	0–2 (1)	0	0–1 (1)	0	11 (1)	40 (1)
	Aspergillus niger JN256017.1.1705	0–1 (1)	0	0	0	0–1 (1)	0	0	0	6 (1)	7 (1)
	Aspergillus JF719077.1.1233	0–1 (1)	0	0	0	0	0	0	0	1 (1)	2 (1)
	Aspergillus niger AY256260.1.1229	0–1 (1)	0	0	0	0	0	0	0	1 (1)	2 (1)
	Candida NR OTU983	0–17 (3)	0–15 (1)	0–95 (7)	0–9 (1)	0	0	0–4 (1)	0	0	0
	Paecilomyces AB015768.1.1686	0–2 (1)	0–2 (1)	0	0–1 (1)	0–1 (1)	0	0	0	5 (1)	5 (1)
	Stachybotrys chartarum JF797222.1.2154	0	0	0	0	0	0–1 (1)	0	0	1 (1)	1 (1)
	Alternaria alternata FM178261.1.2018	0	0	0–0 (0)	0	0	0	0	0	0	0
	Penicillium EU278606.1.1737	0–1354 (1)	0–82 (2)	0–157 (2)	0–129 (2)	0–229 (1)	1–5372 (2)	0–128 (1)	9–37 (3)	1559 (1)	0
	Penicillium (food metagenome) JN624277.1.1430	0–16 (1)	0–2 (1)	0–1 (2)	0	0–1 (1)	0–37 (1)	0–1 (1)	0	17 (1)	0
	Penicillium AB293968.1.1585	0–1 (1)	0	0–1 (2)	0–1 (1)	0–2 (1)	0–9 (1)	0–1 (1)	0	4 (1)	0
	Penicillium AB245443.1.1745	0–1 (1)	0	0–1 (1)	0	0	0–4 (1)	0	0	0	0
	Penicillium KP027014.1.1579	0–1 (1)	0	0	0	0	0–7 (1)	0	0	4 (1)	0
	Penicillium AF454063.1.1667	0–1 (1)	0	0	0–1 (1)	0	0–5 (1)	0–2 (1)	0	5 (1)	0

Genera not found: POTENTIAL PATHOGENS: *Naegleria, Giardia, Blastocystis, Acanthamoeba, Cryptosporidium, Babesia, Cyclospora, Isospora, Toxoplasma, Entamoeba;* WORMS/CONTAMINATION INDICATORS: *Dientamoeba, Enterobius, Ascaris;* AMOEBA HOSTS: *Acanthamoeba, Vahlkamphia;* FUNGI WITH POSSIBLE HEALTH EFFECTS: *Exophiala, Fusarium, Mucor, Rhizopus, Trichoderma, Chaetomium, Aureobasidium, Bauveria, Botrytis, Cladosporidium, Epicoccum, Purporeocillium, Sarocladium.*

NR = new reference; NCR = new cleanup reference, N = number of samples.

Table values: minimum and maximum reads per sample, number of positive samples in brackets. All samples from location 2

Table S11. Free-living amoeba and potentially pathogenic eukaryotes in biofilms.

		Water meter biofilms										Pipeline biofilms	
		A		B		C		D		E		D	
		DNA (N = 2)	RNA (N = 2)	DNA (N = 2)	RNA (N = 2)	DNA (N = 1)	RNA (N = 2)	DNA (N = 1)	RNA (N = 1)	DNA (N = 2)	RNA (N = 3)	DNA (N = 3)	RNA (N = 3)
Free living amoeba	*Balamuthia* (uncultured eukaryote) NCR OTU167741	0	0	0	0	0	0	0	0	0	0	0	0
	Vermamoeba vermiformis NR OTU494	0–9 (1)	2–8 (2)	0	0	0	0–1 (1)	0	0	0	0	2–27 (3)	28–50 (3)
	Vannella epipetala NR OTU56	0	0	0	0–5 (1)	0	0	0	0	0	0	0	0
	Vannella EF032800.1.1760	0	0	0	0	0	0	70 (1)	562 (1)	0	0	0–4 (2)	0–19 (2)
	Vannella NR OTU156	0	0	0	0	0	0	0	0	0	0	0	0
	Vannella epipetala NCR OTU162079	0	0	0	0	0	0	0	0	0	0	0	0
	Vannella NCR OTU11860	0	0	0	0	0	0	32 (1)	1 (1)	0	0	0	0
	Vannella (uncultured eukaryote) NCR OTU186593	0	0	0	0	0	0	51 (1)	40 (1)	0	0	0	0
Fungi	*Aspergillus niger* NR OTU1061	0	0	0	0	78 (1)	0–341 (1)	0	0	0	0	0–1 (1)	0
	Aspergillus niger JN256017.1.1705	0	0	0	0	0	0	0	0	0	0	0	0
	Aspergillus JF719077.1.1233	0	0	0	0	1 (1)	0–1 (1)	0	0	0	0	0	0
	Aspergillus niger AY256260.1.1229	0	0	0	0	0	0	0	0	0	0	0	0
	Candida NR OTU983	0	0	0–2 (1)	0–3 (1)	0	0	0	0	0–53 (1)	0–2 (1)	0	0
	Paecilomyces AB015768.1.1686	0	0	0	0	1 (1)	0–5 (1)	0	0	0	0	0	0
	Stachybotrys chartarum JF797222.1.2154	0	0	0–1 (1)	0	2 (1)	0–4 (1)	3 (1)	0	0	0	0	0
	Alternaria alternata FM178261.1.2018	0	0	0	0	0	0	1 (1)	0	0	0	0	0
	Penicillium EU278606.1.1737	0	1–416 (2)	0	0–1 (1)	226 (1)	9–73 (2)	11 (1)	8 (1)	2–9 (2)	1–8 (2)	21–42 (3)	0–2 (1)
	Penicillium (food metagenome) JN624277.1.1430	0	0–6 (1)	0	0	4 (1)	0	1 (1)	0	0	0	0	0
	Penicillium AB293968.1.1585	0	0–2 (1)	0	0	1 (1)	0	0	0	0	0	0	0
	Penicillium AB245443.1.1745	0	0–3 (1)	0	0	0	0	0	0	0	0	0	0
	Penicillium KP027014.1.1579	0	0	0	0	0	0	0	0	0	0	0	0
	Penicillium AF454063.1.1667	0	0–1 (1)	0	0	0	0	0	0	0	0	0	0

Genera not found: POTENTIAL PATHOGENS: *Naegleria, Giardia, Blastocystis, Acanthamoeba, Cryptosporidium, Babesia, Cyclospora, Isospora, Toxoplasma, Entamoeba;* WORMS/CONTAMINATION INDICATORS: *Dientamoeba, Enterobius, Ascaris;* AMOEBAE HOSTS: *Acanthamoeba, Vahlkamphia;* FUNGI WITH POSSIBLE HEALTH EFFECTS: *Exophiala, Fusarium, Mucor, Rhizopus, Trichoderma, Chaetomium, Aureobasidium, Bauveria, Botrytis, Cladosporidium, Epicoccum, Purporeocillium, Sarocladium.*

NR = new reference; NCR = new cleanup reference, N = number of samples.

Table values: minimum and maximum reads per sample, number of positive samples in brackets.
