# Supplementary material for: Active eukaryotes in drinking water distribution systems of ground and surface waterworks
Source: Microbiome. 2019 Jul 3;7:99. doi: 10.1186/s40168-019-0715-5 (PMC6610866; doi:10.1186/s40168-019-0715-5)
Supplement: Supplementary file 9 — Eukaryotic community (DNA) canonical correspondence analysis (CCA) (Figure S11.) (PDF 454 kb) [file 40168_2019_715_MOESM9_ESM.pdf]

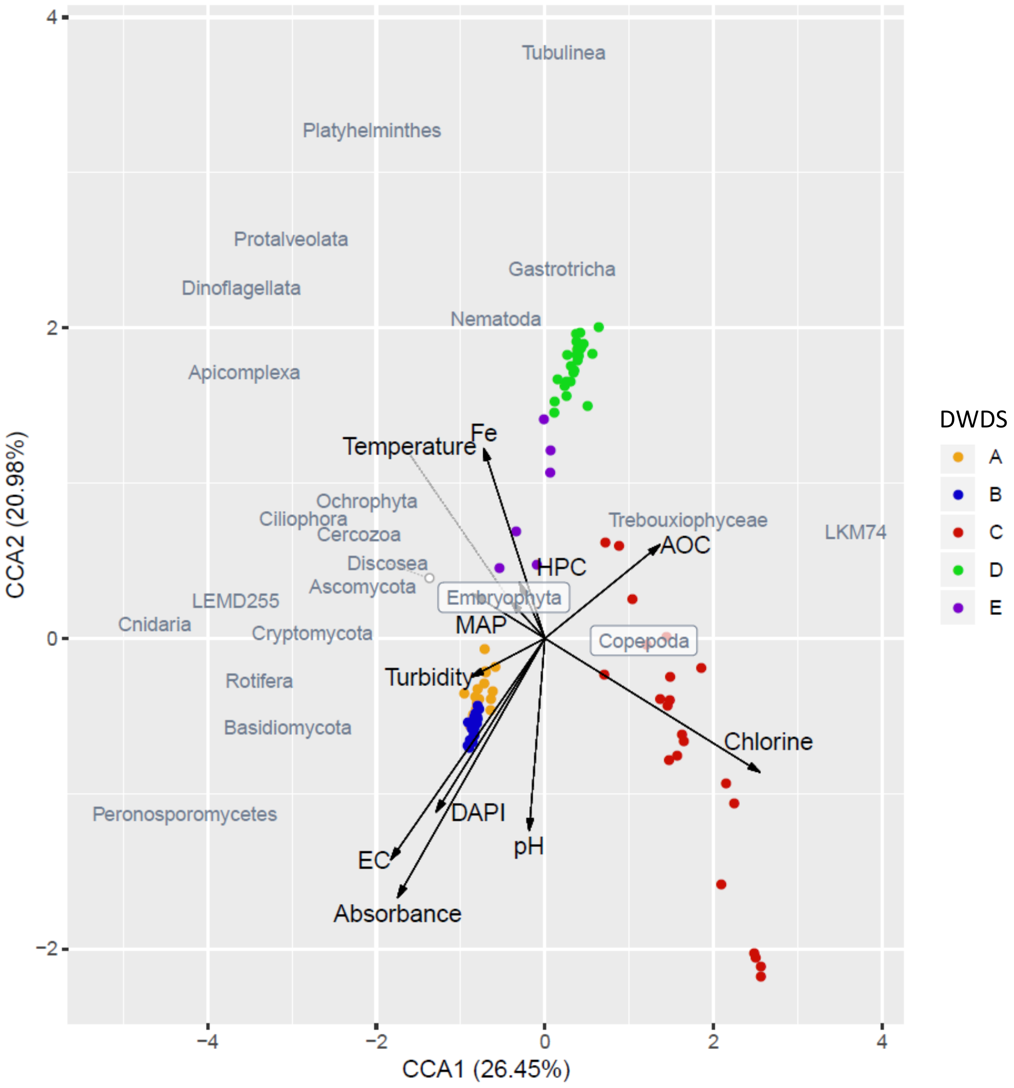

Fig. S11. Total (DNA) eukaryotic community and representative taxa in association with water physico-chemical and microbiological parameters. Explanation rates for CCA1 and CCA2 axes in Canonical Correspondence Analysis (CCA) are in brackets. Each point represents eukaryotic community of one sample colored by waterworks and grey text indicate most abundant eukaryotic taxa (overlapping taxa marked with white circles (exact location) or transparent boxes for clarification). Representative taxa per kingdom: *Amoebozoa* (LKM74, LEMD255 and *Discosea*, *Tubulinea* phyla), *Alveolata* (*Dinoflagellata*, *Ciliophora, Apicomplexa* phyla), *Fungi* (*Ascomycota*, *Basidiomycota*, *Cryptomycota* phyla), *Rhizaria* (*Cercozoa*), *Metazoa* (*Nematoda*, *Copepoda*, *Cnidaria*, *Rotifera*, *Platyhelminthes* phyla), *Chloroplastida* (*Embryophyta, Trebouxiphyceae* class), *Stramenopiles* (*Peronosporomycete*s, *Ochrophyta* phyla). Details of CCA available*

*CCA ANALYSIS DETAILS:

For more clear data representation, the results of the canonical correspondence analysis (CCA), such as biplots (to plot the arrows for the variables) and the species were proportionally scaled.

Firstly, the largest distance from the sites to the origin, $d_{site}^{max} = \max_i \|sites_i\|$, where $sites_i, i = \overline{1,n}$ are the first two components of the CCA results for sites, n is the number of sites and $\|\cdot\|$ is Euclidean norm. There are CCA results for species: $species_j, j = \overline{1,m}$, and loadings: $loadings_k, k = \overline{1,p}$, where m and p are the number of species and environmental variables, respectively.

Secondly, similar values for the species $d_{species}^{max}$ and the variables d_{biplot}^{max} were calculated to provide the normalization of the data so the maximum and minimum values will be equal to those in sites results set.

The scaling coefficients $\gamma_{species}$ and γ_{biplot} (values 1.5 and 0.8, respectively) were adjusted to improve the visualization of the species names and arrows on the plot, increasing the clarity and reducing of the figure or overlapping of the elements.

Finally, the normalization with the adjustment of the scaling was performed according to the following equations:

for sites: $sites^{adj} = sites \cdot \frac{d_{site}^{max}}{d_{species}^{max}} \cdot \gamma_{species}$

for variables: $loadings^{adj} = sites \cdot \frac{d_{site}^{max}}{d_{biplot}^{max}} \cdot \gamma_{biplot}$
